# Numerical and experimental analysis of body armor polymer penetration resistance against 7.62 mm bullet

**DOI:** 10.1016/j.heliyon.2024.e41286

**Published:** 2024-12-17

**Authors:** Gebrewahid Asgedom, Kumlachew Yeneneh, Getu Tilahun, Besufekad Negash

**Affiliations:** a Department of Armament Engineering, College of Engineering, Ethiopian Defence University, Bishoftu, P.O. Box 1041, Ethiopia; b Department of Motor vehicle Engineering, College of Engineering, Ethiopian Defence University, Bishoftu, P.O. Box 1041, Ethiopia

**Keywords:** Polymers, Penetration resistance, ECO-UHMWPE, Impact, Composite materials, Lightweight, Ballistic protection

## Abstract

Advanced materials are crucial for enhancing soldier safety through improved personal body armor. In contrast to conventional Kevlar-epoxy composites, this study examines the ballistic performance of a unique ECO-UHMWPE (Ultra-High Molecular Weight Polyethylene) vest. The aim is to achieve a lightweight design with superior impact resistance, addressing limitations of the current armor used by the Ethiopian Defense Force. A comprehensive finite element analysis (FEA) was conducted using Abaqus explicit dynamics software to simulate the impact of 7.62 × 39 mm projectiles at various ranges (50m, 100m, 150m, and 200m). Material properties, laminate thickness, projectile velocity, and number of layers were systematically varied to assess their impact on vest performance. Results showed that ECO-UHMWPE vests exhibited significantly lower stress values and minimal deformation, particularly at close ranges with high projectile kinetic energy, outperforming Kevlar vests. Ballistic testing validated the FEA, with ECO-UHMWPE vests achieving a 22.44 % weight reduction (3.49 kg, 12 mm thick) compared to traditional Kevlar vests (4.5 kg, 15 mm thick) and offering a 30 % reduction in deformation and a 25 % increase in energy absorption. This analysis demonstrates the potential of ECO-UHMWPE for lighter, more effective body armor, paving the way for next-generation personal protective equipment.

## 1 Introduction

Body armor, often referred to as bulletproof vests or personal protection equipment, is specifically engineered to protect the wearer from ballistic threats such as bullets, shrapnel, and other projectiles. These protective systems are critical in military, law enforcement, and civilian applications, where ensuring the safety of personnel in hostile environments is paramount. Modern body armor consists of multiple layers designed to absorb and dissipate the kinetic energy of projectiles, thereby preventing or mitigating injuries [1]. This protection is essential for safeguarding vital organs like the chest and abdomen, which are the most susceptible to injury from gunfire [2].

The fundamental structure of body armor includes an outer shell, typically made from durable synthetic fabrics, and internal layers made from a range of materials. The internal layers are designed to offer ballistic resistance.

**Table:** Abbreviation Description

API	Armor pricing in the cidery
Al 5083	Aluminum alloy
BFD	Back face deformation
BPI	Ballistic performance index
CFRP	Carbon fiber reinforced polymer composite
ECO	Envi mental conductive
EASK	Enhance armor system kinetics.
EFPs	Explosively formed projectiles
FEM-SPH	Finite element model -smooth particle hydrodynamic
GFRP	Glass fiber reinforced polymer composite
IED	Improvised explosive devices
KFRP	Kevlar fiber-reinforced polymer composite
KE	Kinetic energy
LS -DYNA	Livermore Software Technology Corporation dynamic analysis
VIP	Very important person
UHMWPE	Ultra-high molecular weight polyethylene
UV	Ultraviolet
ECO-UHMWPE	Environmental conductive-ultra high molecular weight polyethylene

and are composed of materials such as metals (e.g., special steel, aluminum), ceramics (e.g., nitrides, carbides, oxides), Kevlar®, fiberglass, ultra-high molecular weight polyethylene (UHMWPE), and advanced polymers with self-healing properties [3],. Each material contributes to the overall energy absorption mechanism, with metals and ceramics providing the ability to blunt or fragment projectiles, while fiber-based materials like Kevlar® and UHMWPE offer flexibility, tensile strength, and energy dissipation through deformation [4,5].

Historically, body armor has evolved from rudimentary designs to sophisticated systems. Early examples of personal protection can be traced back to ancient civilizations, such as the Persians and Greeks, who utilized armor made of multiple layers of linen, while warriors in the Gilbert and Ellice Islands relied on coconut palm fiber for protection until the 19th century [6]. Over time, technological advancements led to the development of metal-based armors, such as a 44 kg suit created in Australia in 1879 from metal scraps, providing protection for the torso, arms, and legs. By World War I, military body armor incorporated hard plates to safeguard the chest and head against shrapnel and bullets [7].

The evolution of body armor has culminated in two primary categories: hard and soft armor [8]. Hard armor relies on rigid materials like metal and ceramic composites for high-impact resistance, making it suitable for scenarios where protection against armor-piercing bullets is required. Soft armor, by contrast, is made from flexible materials and consists of multiple layers of woven or non-woven fibers, offering enhanced comfort and mobility while still providing substantial protection against low-velocity projectiles. These advancements have been driven by the need to balance protection with weight and comfort, crucial factors for soldiers and law enforcement personnel in the field [1,9].

Among the most promising materials for modern ballistic protection is UHMWPE, a type of polyethylene characterized by its high molecular weight and ultra-dense polymer chains. UHMWPE fibers exhibit extraordinary resistance to ballistic impacts, outperforming traditional aramid fibers in terms of energy absorption and impact distribution. ECO-UHMWPE (Environmentally Correct Ultra-High-Molecular-Weight Polyethylene) distinguishes itself from traditional UHMWPE by combining exceptional mechanical properties with enhanced environmental resistance. Unlike ordinary UHMWPE, ECO-UHMWPE offers superior impact resistance and energy absorption, making it highly effective against high-velocity projectiles. Additionally, ECO-UHMWPE contributes to weight reduction and environmental sustainability, key objectives of this study. These characteristics make it a novel choice for lightweight, high-performance body armor systems. This material is lightweight, with a density significantly lower than carbon fibers, and demonstrates exceptional flexibility, which prevents secondary injuries by reducing blunt force trauma. As a result, UHMWPE has become a key component in the design of high-performance ballistic vests, helmets, and other protective equipment. The fibers catch and deform bullets upon impact, spreading the force across a larger area and preventing penetration into the wearer's body [3,10].

### 1.1 Energy absorption mechanism in composite materials

The capacity of body armor to absorb and disperse bullet kinetic energy is what makes it effective. When a bullet impacts the armor, several energy absorption mechanisms are activated, allowing the armor to withstand and mitigate the ballistic threat. These mechanisms include the deformation of the bullet and armor material, fiber fracture, matrix cracking, and delamination within the composite layers [2,11].

Composite materials, widely used in body armor, are composed of a matrix (such as a polymer) reinforced with high-strength fibers (e.g., Kevlar®, UHMWPE). When a projectile strikes the armor, the energy is absorbed through several processes. First, a kinetic energy transfer occurs, where the projectile's momentum is converted into deformation energy in both the projectile and the armor. This is followed by shear plugging, where the projectile penetrates the outermost layers of the armor, and tensile failure of the primary fibers, which absorb significant amounts of energy by stretching and breaking. Matrix cracking occurs in the polymer, distributing the load and further reducing the projectile's velocity [12].

The deformation of the armor material, particularly the fibers, is critical in preventing penetration. As the fibers elongate and fracture, they absorb energy, slowing the projectile. Delamination, or the separation of composite layers, further dissipates the energy by creating additional failure surfaces that absorb impact forces [13]. The combination of these mechanisms provides a complex, multi-faceted defense against ballistic threats, ensuring that the projectile's energy is distributed across a larger area and its velocity is significantly reduced before it can cause harm to the wearer [14].

Recent advancements in ballistic materials, such as the introduction of ECO-UHMWPE, have led to the development of lighter, more durable, and more efficient body armor [15]. ECO-UHMWPE fibers possess enhanced environmental resistance, durability, and ballistic performance, making them ideal for personal protective systems in both military and civilian contexts. These fibers, integrated into multi-layered body armor systems, improve energy absorption while maintaining the lightweight characteristics necessary for operational effectiveness [16].

### 1.2 Penetration resistance mechanism in composite materials

The penetration resistance of body armor is achieved through the interaction of the armor materials with the projectile. When a high-velocity bullet strikes a composite armor system, it induces complex stress waves and material deformation. The bullet's kinetic energy causes lateral and downward displacement of the armor material, leading to localized compression and out-of-plane deformation. This initial impact results in both mode-I (opening) and mode-II (shearing) inter-laminar cracking [17].

In a multi-layered armor system, the front layer, often made of ceramic materials such as alumina or silicon carbide, plays a crucial role in blunting or fracturing the projectile. These hard materials dissipate the initial energy of the impact by breaking the bullet and reducing its momentum [18]. The ceramic layer's high hardness and compressive strength enable it to effectively absorb and disperse the projectile's energy over a larger area, preventing it from fully penetrating the armor. Ceramics are combined with ductile structural components like steel, aluminum alloys, or plastics with fiber reinforcement since they are brittle and can break under extreme stress [19,20].

The backing materials serve to absorb the residual energy from the projectile after it has been blunted or eroded by the ceramic front layer. Fiber-reinforced polymers, in particular, exhibit excellent energy absorption properties due to their low density and high tensile strength [21,22]. These materials undergo a number of failure processes, including as delamination, structural splitting, and fiber fracturing, all of which enhance the armor system's overall effectiveness against infiltration. The armor offers optimal protection against ballistic threats without sacrificing weight or flexibility thanks to the cooperation of the ceramic front layer and the composite backing layers [23,24].

Despite significant advancements in body armor technology, the increasing lethality of modern firearms, such as the widespread use of 7.62 mm bullets, presents new challenges in designing armor that offers both maximum protection and operational mobility [25]. Traditional materials like metal plates provide excellent protection but are often too heavy for extended use in dynamic combat situations. Conversely, soft body armor, while lightweight, may not always provide sufficient protection against high-velocity projectiles [26,27].

The development of advanced polymers like UHMWPE, along with composite materials combining ceramics and fiber-reinforced polymers, offers the potential to bridge this gap. However, a comprehensive understanding of the penetration resistance of these materials against 7.62 mm bullets is still required. This study, therefore, seeks to address this gap by conducting a detailed numerical and experimental analysis of the penetration resistance of polymer-based body armor against 7.62 mm ballistic threats. By integrating finite element analysis (FEA) with real-world experimental testing, this research aims to optimize the design and material composition of body armor systems to improve their protective capabilities while minimizing weight and bulk. The outcomes of this study will advance to the design of more efficient, lightweight body armor systems capable of withstanding modern ballistic threats, ultimately enhancing the safety and operational efficiency of military and law enforcement personnel.

## 2 Material and methodology

This section 2 outlines the design, Material selection, and analytical methodology used to evaluate the penetration resistance of a composite-based bulletproof vest. This study focuses on developing a lightweight, high-performance armor solution by optimizing the protection area and reducing the weight of existing ceramic-based body armor, which is currently employed by the logistics department of the Ethiopian Defense Force Both finite element analysis (FEA) and simulated trials are used to support the design process in order to evaluate how well ECO-UHMWPE and Kevlar-epoxy composites perform against 7.62 mm ballistic threats.

### 2.1 Materials

This research focuses on two primary materials: Kevlar-epoxy composites and ECO-UHMWPE, both of which are widely recognized for their effectiveness in ballistic protection applications. Kevlar-epoxy composites are chosen for their exceptional tensile strength, lightweight properties, and impressive resistance to impact and abrasion. Kevlar fibers, known for their ability to absorb significant amounts of energy upon impact, serve as the primary load-bearing component of the composite. The epoxy matrix, on the other hand, provides structural integrity by bonding the Kevlar fibers together and enhancing the material's durability. This synergy between the fibers and the matrix allows for effective dispersion of impact energy, significantly reducing the risk of penetration. Kevlar-epoxy composites are particularly advantageous in ballistic protection because they offer a high degree of protection while minimizing the weight of the body armor, making them suitable for military and law enforcement use.

ECO-UHMWPE is another high-performance material known for its superior strength-to-weight ratio and excellent impact resistance. Unlike traditional materials, ECO-UHMWPE exhibits outstanding energy absorption capabilities, making it highly effective against high-velocity ballistic threats such as 7.62 mm bullets. The polymer's low density combined with its remarkable ability to withstand deformation under stress makes it an ideal choice for lightweight body armor, helmets, and vehicle armor. ECO-UHMWPE's molecular structure allows for efficient energy dissipation, absorbing and distributing the force of impact across a wider surface area, which helps prevent penetration while maintaining a lightweight and flexible design.

In this study, the primary focus is to evaluate and compare the ballistic performance of Kevlar-epoxy composites and ECO-UHMWPE in resisting penetration from 7.62 mm bullets. By analyzing their energy absorption capabilities, structural integrity, and potential for use in lightweight armor systems, this research aims to determine the most effective material for body armor that offers superior protection without compromising mobility and comfort.

### 2.2 Methodology

This study employs a combined approach of finite element analysis (FEA) and experimental testing to evaluate the ballistic performance of Kevlar-epoxy composites and ECO-UHMWPE against 7.62 mm bullets. The FEA is conducted using ABAQUS/CAE Explicit dynamics, a robust tool for simulating high-velocity impacts. First, a detailed finite element model of the bulletproof vest is created, incorporating the precise material properties of both Kevlar-epoxy composites and ECO-UHMWPE. Realistic boundary conditions are applied to replicate the actual impact scenario, including the projectile's velocity and the vest's structural constraints. The simulation aims to predict how these materials behave under ballistic impact, focusing on energy absorption, deformation, and potential penetration. Alongside the simulations, experimental tests are carried out to validate the numerical results. Bullet impact tests are performed on sample panels of Kevlar-epoxy and ECO-UHMWPE composites, measuring critical performance metrics such as bullet deformation, residual velocity, and vest damage. The combination of FEA and experimental testing ensures a comprehensive analysis of the materials' protective capabilities, providing insights into their effectiveness in real-world ballistic protection applications.

### 2.3 Fundamental principles of impact dynamics

Impact events are generally analyzed using three basic principles, whether in numerical simulation, ballistics modeling, or stress wave propagation. The conservation of mass, conservation of momentum, and conservation of energy are these three concepts. The conservation of mass, expressed in Equation (1), ensures that the total mass in a system remains constant:

(1) ∫vρdv=const

For a closed system of n masses (Mi) with no external forces, the conservation of momentum, as shown in Equation (2), governs the relationship between forces and the change in momentum. Based on Newton's second law, Equation (3) describes the rate of change of momentum with respect to time. When Equation (3) is integrated over time, the impulse-momentum relationship, detailed in Equation (4), is obtained:

(2) $$\sum_{i=1}^{n}M_{i}V_{i}=const$$

(3) $$F=m\frac{dV}{dt}$$

(4) $$I=\int Fdt=\int Mdv=MV_{f}-MV_{i}$$

where I is impulse, F is the impacting force, M is the mass of the object, V_f_ is the final velocity, and V_i_ is the initial velocity. Conservation of energy, as expressed in Equation (5), states that the energy in a system remains constant over time:

(5) $$\sum_{j}E_{i}+\sum_{j}\frac{1}{2}\rho v_{i}^{2}=\sum_{j}E_{f}+\sum_{j}\frac{1}{2}\rho v_{f}^{2}+W$$

These fundamental principles are integral to modeling impact phenomena in Abaqus Explicit Dynamics, where inputs such as the bullet's mass and the material's density are used to predict the system's behavior under high-velocity impacts.

#### 2.3.1 Formulation of the problem

Transient deformations of a continuous body, such as the bulletproof vest and projectile, are governed by the Equation of motion expressed in Equation (6):

(6) $$\begin{array}{l} \rho \dot{s}=\sigma_{ij,j}+\rho \\ i=1,2\ldots \end{array}$$

Where: ρ = density, s = displacement of a material points along the x-axis.

$\sigma_{ij,j}=\frac{\partial \sigma \text{ij},}{\partial x_{j}},\sigma_{ij}$, the Cauchy stress tensor, $b_{i}$ = body force per unit mass a superimposed

where ρ is the material density, si is the displacement of material points along the xx-axis, σij represents the Cauchy stress tensor, and bi is the body force per unit mass. The term σij,j refers to the spatial derivative of the stress tensor, and a repeated index implies summation over its range. A dot over a variable indicates the material time derivative. For the purposes of this analysis, it is assumed that the bulletproof vest is initially at rest, free from residual stresses, and has no displacements, while the projectile possesses a non-zero initial velocity and is stress-free before impact. The effects of gravitational forces on the deformation are neglected, as the body force bi is considered zero in this context. Equation (6) considers the three-dimensional stress state in composite laminates. To simplify, the analysis focuses on x = 1, representing the primary impact direction. This assumption emphasizes uniaxial modeling, which captures the dynamic stress concentration critical to penetration resistance. While longitudinal wave velocity (Equation (7)) is the primary focus, transverse, Rayleigh, and plate waves also contribute to broader stress distribution but are secondary to the study's primary objectives.

As described by Lyons, the speed of the plastic wave and the transverse wave velocity in ultra-high molecular weight polyethylene (UHMWPE) materials, along with the corresponding plastic strain, are derived from Equations (1), (2). Assuming a Poisson's ratio of ν = 0.3, these relationships simplify to Equation (7), which gives the plastic wave speed as a function of the material's modulus of elasticity and density. Additionally, the relationship between the transverse wave speed and plastic strain is expressed in Equation (8), where the strain is a function of material constants and the plastic strain. These Equations are fundamental in understanding the dynamic response of UHMWPE under impact.

(7) $$c_{p}=\sqrt{\frac{E}{\rho }}$$

(8) $$C_{T}=C_{p}\sqrt{\frac{\varepsilon_{p}}{1+\varepsilon_{p}}}$$

### 2.4 Experimental evaluation of kevlar-based bulletproof vest against 7.62 mm bullets

In this study, a thorough experimental evaluation was carried out to examine the ballistic performance and mechanical resilience of a Kevlar-based bulletproof vest. The vest, with dimensions of 250 × 300 mm and a thickness of 15 mm, underwent two distinct testing phases: ballistic resistance testing using 7.62 mm projectiles and non-ballistic mechanical impact assessments to observe the material's behavior under controlled mechanical loads.

The first phase involved subjecting the vest to impacts from 7.62 × 39 mm bullets fired from varying distances (50 m, 100 m, 150 m, and 200 m) at the Gafat Armament Engineering Industry. The objective was to replicate real-world combat conditions and evaluate the vest's capacity to stop projectiles at different ranges. Results showed complete penetration of the vest at 50 m and 100 m, indicating a complete failure in protection at these short distances. However, at 150 m and 200 m, partial penetration occurred, suggesting significant blunt force trauma and a heightened risk of internal injury due to the transfer of kinetic energy. Visual inspections of the damage revealed circular cracks and interlaced fractures, with the most pronounced deformation observed at the closer ranges.

To further quantify the vest's performance, ballistic limit velocity tests were conducted to determine the minimum velocity needed for full penetration and the maximum velocity at which the bullet lost all residual speed. The 7.62 mm projectile, with an initial muzzle velocity of 838 m/s, showed a 10 % reduction in velocity for every 50 m of travel, accounting for factors such as air resistance. These tests were crucial in gathering data on the energy absorption capabilities of the vest, enhancing the understanding of its ballistic resistance across a range of distances.

In the second phase, mechanical impact tests were performed at the Armament Engineering Department Lab, Defense Engineering College. These tests involved dropping weights from controlled heights onto undamaged sections of the vest to simulate non-ballistic mechanical forces. The objective was to assess the material's durability, elasticity, and its ability to withstand mechanical stresses without incurring permanent damage. The recorded results, including photographs, visually illustrated the vest's capacity to endure non-ballistic forces, further contributing to the evaluation of its overall performance.

The findings from both the ballistic and mechanical tests underscore the limitations of the Kevlar-based vest in providing adequate protection against high-velocity impacts at close range. While the vest demonstrated some level of resistance at longer distances, the results suggest a need for further material enhancements or structural improvements to optimize protection without compromising on weight and mobility. These insights are crucial for guiding the future development of advanced body armor systems capable of withstanding modern battlefield threat.

**Fig. 1:**
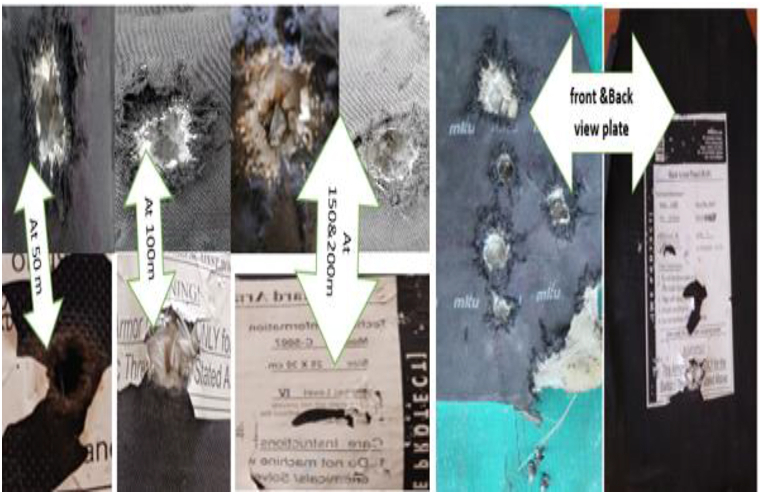
Front and back face of Impact trial by 7.62 mm projectile on existing vest.

The impact velocity (V_amp_) at each range can be calculated using Equation (9), where the factor 0.9 accounts for a 10 % reduction in velocity due to decelerating forces. The residual velocity (V_residual_) is determined by the material's resistance and is calculated using Equation (10). Additionally, the kinetic energy (K.E.) of the bullet is computed using Equation (11), which relates the kinetic energy to the mass of the bullet (Mb) and the muzzle velocity (V), with the bullet mass set at 10.8 g and the muzzle velocity at 838 m per second. Finally, as shown in Equation (12), the kinetic energy of the bullet is calculated to be 3792.1176 J, providing a quantifiable measure of the energy transferred during impact. The experimental results, including the perforation and non-perforation outcomes, are detailed in Table 1, and the impact marks on the vest are depicted in Fig. 1.

(9) $$V_{impact}=V{\left(1-decay\;factor\right)}^{n}$$

(10) $$V_{residual}=V_{impact}\left(1-resist\;factor\right)$$

(11) $$K.E=\frac{1}{2}M_{b}V^{2}$$

(12) $$K.E=\frac{1}{2}\left(0.0108\right){\left(838\right)}^{2}=3792.1176J$$

**Table 1:** Experimental data observed from firing test on the body armor vest for Kevlar.

Thickness (mm)	Range in(m)	Impact Velocity (m/s)	Residual velocity (m/s)	Perforated(P) or Non-perforated(N)	Front Dia (mm)
15	50	754.2	83.8	Perforated	22
15	100	678.78	75.42	Perforated	20
15	150	610.9	67.88	12.9 mm (N-P)	19
15	200	549.81	61.2	13.8 mm (N-P)	18

#### 2.4.1 Impact testing on Kevlar vest under varied heights

In the second phase of our experiment, we performed impact tests on undamaged sections of the Kevlar bulletproof vest using a calibrated impact testing machine. The machine, weighing 3.741 kg and raised to a height of 1 m, was used to simulate various impact conditions. To assess the material's response to different energy levels, the testing involved releasing the mass from four predetermined heights: 0.45 m, 0.6 m, 0.8 m, and 1 m. Each height corresponded to a specific impact energy, representing the potential energy converted into kinetic energy upon striking the vest. The test samples were firmly secured to maintain consistent positioning throughout each trial. The resulting forces from the impacts were measured and documented, with the results summarized in Table 2, offering valuable insights into the vest's performance under non-ballistic impact conditions.

**Table 2:** Drop weight impact test results.

Trial	t in sec)	H in (m)	D_h_ (mm)	Observation
1	0.44	0.45	1.2	A. Very small dot on the piece
2	0.47	0.60	1.21	B. A small hole created in the piece
3	0.53	0.80	2.51	C. A Larger hole was created on the piece
4	0.60	1	2.54	D. A very large hole was created but not penetrated throughout the piece

**Fig. 2:**
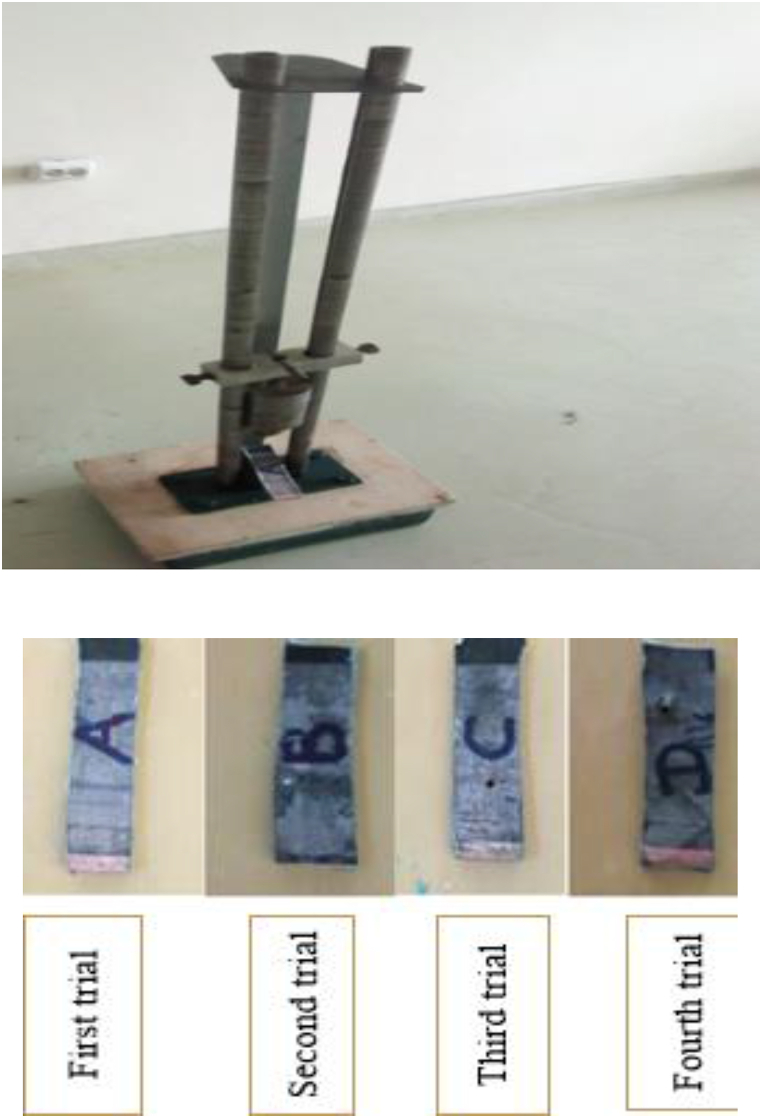
Shows the setup for the drop weight experiment of the impact test machine.

Fig. 2 presents the experimental setup for the drop weight impact testing conducted on the Kevlar bulletproof vest. These tests provided detailed data regarding the vest's resistance to both ballistic and non-ballistic impact forces, facilitating an in-depth evaluation of its protective performance and identifying potential areas for improvement. The results, as illustrated by the images of the tested samples, show a clear relationship between the increase in impact height and the degree of damage sustained by the vest. This correlation highlights how the severity of the damage escalates with higher impact energies, further informing the optimization of the vest's protective capabilities. As the impact height increased from 0.45 m to 0.6 m, and further to 0.8 m and 1 m, the damage intensified, with the samples showing progressively larger perforations. The first trial produced a minor indentation, while subsequent trials resulted in increasingly larger holes, indicating heightened material stress under greater impact forces. The kinetic energy imparted during each impact was calculated using the principle of conservation of energy, which posits that energy cannot be created or destroyed but is instead transferred between forms.

In this context, the potential energy due to the height of the mass was converted into kinetic energy upon impact with the vest. The energy involved in each trial was calculated using Equation (16), we see that the potential energy is entirely converted into kinetic energy during the impact, providing essential insights into the vest's ability to absorb and dissipate energy under varying conditions.

(13) $$E_{i}=Mgh$$

(14) $$E_{f}=\frac{1}{2}MV^{2}$$

(15) $$Mgh=\frac{1}{2}V^{2}$$

(16) $$2gh=V^{2},V=\sqrt{2gh}$$

#### 2.4.2 Design considerations and assumptions for ECO-UHMWPE bulletproof vest

The design considerations and assumptions for the ECO-UHMWPE bulletproof vest focus on enhancing material durability, environmental resistance, weight optimization, and ballistic performance. These factors are essential to ensure that the vest provides effective protection while maintaining comfort and mobility for the wearer. In analyzing the normal impact response of composite bulletproof vests along the Z-axis, it is assumed that a 10.8-g rigid projectile, traveling at 838 m/s, impacts the vest perpendicularly. The polyolefin resin composite laminate is oriented with the 0° ply facing the projectile. Several assumptions Equation (13) and Equation (14), where (M) represents the mass, (g) is the gravitational acceleration, (h) is the height, and (V) is the velocity. By equating the potential energy to the kinetic energy, as shown in Equation (15) and Equation underpin this design: the impact is modeled as uniaxial, focusing only on the force in the direction of the projectile, and the shape of the bullet is disregarded in the calculation of impact force to simplify the analysis. Additionally, energy losses from friction, delamination, and uneven failure mechanisms across the composite's thickness are not considered for this streamlined evaluation. The 7.62 × 39mm AK-47 projectile was selected due to its known lethality against bulletproof vests, making it an appropriate choice for testing penetration resistance. The pressure values derived from energy-work and impulse-momentum Equations, outlined in Equations (17), (18), align with observed ballistic data, forming a solid foundation for further analysis and validation of the vest's protective capabilities.

(17) $${\left[0^{0}/30^{0}/\pm 45^{0}/60^{0}/90^{0}\right]}_{S}$$

(18) $$\begin{array}{l} W=FX\\ F=Ma\\ W=△KE \end{array}$$

These findings are further supported by the specifications outlined in Table 3 and the visual evidence provided in Fig. 1. The impulse-momentum Equation, provided in Equations (19), (20), is used to calculate the impact force, which is derived from the linear momentum change. The change in velocity, assuming no penetration of the target (i.e., V_f_ = 0), is given in Equation (21). From these calculations, the force with which the bullet impacts the bulletproof vest is approximated as 90.504 KN along the x-axis, indicating the intensity of the impact force applied to the material during testing.

**Table 3:** Specifications of AK-47machin gun.

Class	Weapon	Caliber	M_b_ (g)	Test range(s)	Muzzle v
Assault Rifle	AK-47	7.62 ×3 9 mm	M = 10.8	50–200 m	838 m/s

**Table 4:** Comparison between ECO-UHMWPE and Kevlar.

Property	ECO-UHMWPE	Kevlar	Units
Projectile Mass	9.6–10.8	10.8 g	g
Projectile Velocity	760–838	760–838	m/s
Projectile Energy	2179	2179	J
Projectile Young's Modulus	117	117	GPa
Projectile Density	8960	8960	kg/m³
Vest Density	970	1446	kg/m³
E₁ Young's Modulus	16091.4	12021.8	GPa
E₂ Young's Modulus	13022		GPa
E₃ Young's Modulus			GPa
Shear Modulus G₁₂	0.356.3	0.4131.19	GPa
Shear Modulus G₂₃	0.4131.19		GPa
Shear Modulus G₁₃	0.356.27		GPa
Poisson's Ratio ν₁₂ = ν₁₃	0.420.35-	0.400.33-	
Poisson's Ratio ν₂₃			
Longitudinal Tensile Strength	3.6	3.5	GPa
Longitudinal Compressive Strength	1.9	1.1	GPa
Transverse Tensile Strength	2.8	0.45	GPa
Transverse Compressive Strength	1.6	0.15	GPa
Longitudinal Shear Strength	0.44105		MPa
Transverse Shear Strength	0.2398		MPa
Longitudinal Tensile Fracture Energy	1908.42		kJ/m^2^
Longitudinal Compressive Fracture Energy	1408.42		kJ/m^2^
Transverse Tensile Fracture Energy	0.608.43 kJ/m^2^		kJ/m^2^
Transverse Compressive Fracture Energy	1.858.42 kJ/m^2^		kJ/m^2^
Energy Absorbed	3200 J	1500 J	J

Note: All values in Table 4 were validated against experimental and literature data. Units such as GPa, MPa, and kg/m³ are consistent with standard material property definitions and numerical inputs for finite element analysis.

From the impulse-momentum Equation:

(19) $$I=\int Fdt=\int Mdv=MV_{f}-MV_{i}$$

(20) $$F_{i}\times t_{impact}=MV_{f}-MV_{i}$$

(21) $$F_{i}\times t_{impact}=MV_{f}-MV_{i}$$

Designing a bulletproof vest using ECO-UHMWPE (Environmentally Correct Ultra-High-Molecular-Weight Polyethylene) necessitates a thorough analysis of the forces and stresses that occur during ballistic impacts. This material is recognized for its exceptional strength-to-weight ratio and impressive energy absorption capabilities, which are essential for advanced ballistic protection. When a projectile impacts the vest, it generates substantial stress that must be distributed efficiently across the material to prevent penetration. The ability of ECO-UHMWPE to absorb and dissipate kinetic energy is crucial for ensuring the vest's overall performance. By optimizing the design to manage stress distribution effectively, the vest can provide superior protection while minimizing deformation and reducing the risk of trauma to the wearer. The stress distribution and energy absorption properties of ECO-UHMWPE are pivotal in enhancing the vest's ability to stop high-velocity projectiles, ensuring both safety and mobility for the user.

(22) $$\sigma_{t}=\frac{F}{A}$$

(23) $$\tau =\frac{F}{2A}$$

(24) A=L×W

The stress distribution in the vest is calculated using Equation (22) and Equation (23), where stress (σ) is defined as the force per unit area. The impact force applied by a bullet on the armor surface along the x-axis is approximately 1,206,720 N. The bulletproof vest consists of two panels (front and back), each with dimensions of 250 mm by 300 mm and a thickness of 12 mm. The area of each panel is given by Equation (24). The dimensions for the Kevlar panel are 250 mm × 300 mm × 15 mm, while the ECO-UHMWPE panel measures 250 mm × 300 mm × 12 mm, with a total of 24 plies. To calculate the mass of the body armor based on its given dimensions and density, the volume of the panel is calculated as described in Equation (25).

(25) V=A×t

The normal stress applied on the vest is given by the relation σ = F/A, resulting in an approximate normal stress of 3792.889 N/m^2^. The weight of the vest is determined using the density and volume relationship ρ = M/V where ρ is the density of the material. In this case, the bulletproof vest is made from Dyneema® Spectra 1000 polyethylene fiber, which has a density of 970 kg/m³.The mass of the newly designed Dyneema® bulletproof vest is calculated as M = ρ V, and the final mass of the vest is approximately 3.49 kg.

#### 2.4.3 Laminate thickness and number of laminas

To calculate the thickness of a single-ply lamina, we assume that the total thickness of the laminate is 12 mm. Given that the laminate consists of 24 plies, with the ply orientation defined earlier as (0°300/±45°/60°/90°) s, the thickness of a single lamina can be determined by dividing the total thickness by the number of plies. This relationship is outlined in Equation (26).

(26) $$thickness\;of\;\sin\;gle\;la\;\min\;a=\frac{12mm}{24ply}=0.5mm$$

The Kevlar bulletproof vests currently in use by the Ethiopian Defense Force weigh 9 kg (distributed as 2 panels of 4.5 kg each) and have a thickness of 15 mm. Ballistic tests reveal that these vests fail to withstand impacts from 7.62 × 39 mm projectiles, which possess a kinetic energy exceeding 92.1 J, resulting in perforation at distances of 50 m, 100 m, and 150 m. In contrast, the newly developed ECO-UHMWPE vests offer significant improvements, weighing only 3.49 kg and featuring a reduced thickness of 12 mm. Despite this reduction in weight and profile, the new vests do not sacrifice protection. Instead, they enhance both mobility and comfort—critical factors in active combat scenarios. Comparative ballistic tests indicate that the ECO-UHMWPE vests provide superior resistance to 7.62 × 39 mm projectiles at high kinetic energies, effectively halting penetration and offering enhanced protection with a considerable reduction in weight.

### 2.5 Computational models

This study uses computational modeling and simulation to evaluate the penetration resistance of Kevlar and ECO-UHMWPE composite material armor by 7.62 × 39 mm kinetic energy. The primary objectives are to accurately predict the material's impact resistance performance, optimize design configurations, reduce development time and costs, and ensure safety and reliability. The study analyzes how both vests' fibers respond to ballistic impacts, analyzes stress distribution and energy absorption and dissipation, and validates computational models against experimental data to ensure the final product meets stringent safety standards and consistently performs in real-world scenariosTable.4 The input parameters during numerical analysis for ECO-UHMWPE and Kevlar epoxy bulletproof vest with a bullet.

#### 2.5.1 Simulation test

**Fig. 3:**
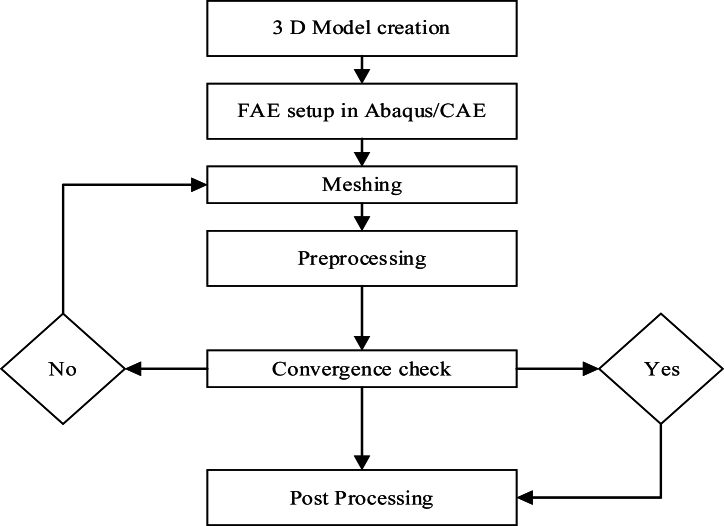
Road map of the methodology.

The simulation process begins with obtaining a 3D model, which serves as the foundation for subsequent analysis. Following the creation of the model in Abaqus, the next stage involves meshing, where the mesh size and number of elements are carefully defined. A convergence analysis is then conducted to determine whether the chosen mesh size provides accurate results. If convergence is achieved, the simulation progresses to the post-processing phase, where results are analyzed. However, if convergence is not obtained, adjustments to the meshing parameters are necessary to ensure reliability. All outputs from the simulation undergo detailed post-processing analysis to validate the findings. The overall methodology is outlined in Fig. 3, which illustrates the sequential steps involved in the simulation workflow.

The shield analyzed in this study is a composite system consisting of multi-layered ECO-UHMWPE laminates with plies oriented at (0°/30°/±45°/60°/90°). The total thickness is 12 mm, optimized for ballistic resistance. The finite element analysis (FEA) incorporates a projectile modeled as a spherical impactor, approximating a 7.62 mm bullet. Boundary conditions include fixed constraints at the back face (u1 = u2 = u3 = 0) to simulate realistic operational scenarios. Meshing with a size of 0.002 mm produced 162,489 elements and 323,037 nodes, ensuring computational precision. Simulations, performed in ABAQUS/CAE, assess stress, strain, and deformation at various impact ranges (50 m–200 m). The finite element model comprises two primary components: the bulletproof vest and the projectile. Various parameters were calibrated for the analysis, with Dyneema® Spectra 1000 polyethylene fiber utilized for the vest and structural steel for the projectile. The Finite Element Analysis (FEA) simulation was conducted using ABAQUS/CAE 2024 software, facilitating model creation, monitoring of the analysis progress, and evaluation of the results.

**Fig. 4:**
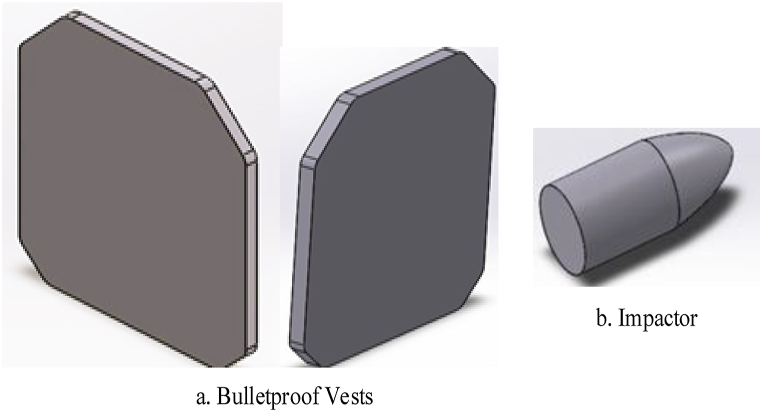
[a, b]3D model for bulletproof vests and bullet.

A typical Finite Element Analysis (FEA) process is divided into three main phases: pre-processing, simulation, and post-processing. In the pre-processing phase, an input file is created within the ABAQUS/CAE environment to define the physical problem to be analyzed. During the simulation phase, the problem is fully defined and analyzed. The model consists of two types of deformable laminate plates: one representing the current vest with dimensions of 250 mm × 300 mm x 15 mm, and another for the new design, sized at 250 mm × 300 mm x 12 mm. The projectile is modeled as a 7.62 mm spherical impactor, as depicted in Fig. 4 (a, b). Both the existing and new vests are modeled using ABAQUS/CAE 2024, with a stacked laminate configuration following a (0°/30°/±45°/60°/90°) symmetry. In the post-processing phase, the results from the analysis are stored in an output file and analyzed using the visualization module of ABAQUS/CAE 2024.

**Fig. 5:**
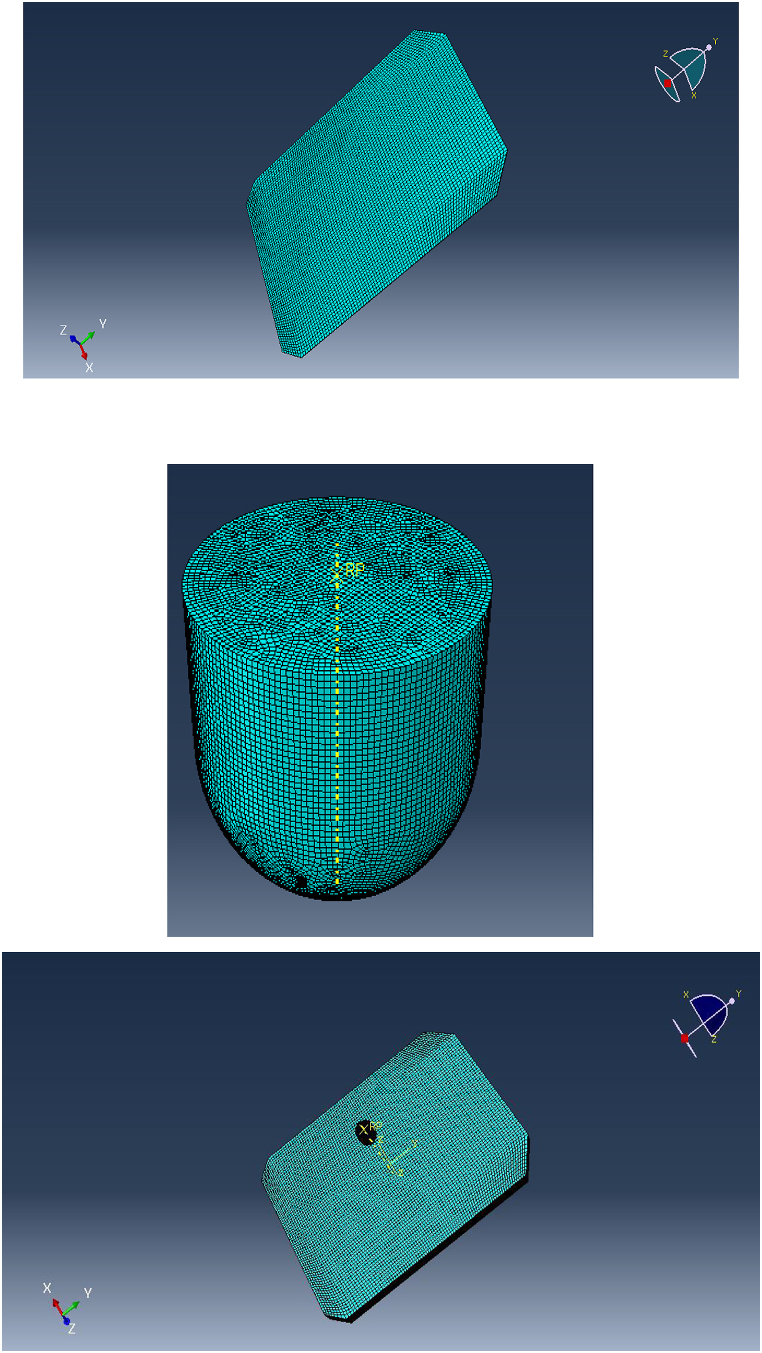
Meshing of bullet and bulletproof vest.

To accurately simulate the composite layers of the bulletproof vest and the impactor, a discretization process is conducted using the Mesh module, as shown in Fig. 5. Both the composite plate and the impactor are meshed with an appropriate element size that strikes a balance between computational efficiency and the required accuracy. A mesh convergence study was performed to verify that the chosen mesh size accurately captured the dynamics of the impact event. A mesh size of 0.002 was selected, resulting in 162,489 elements and 323,037 nodes in the mode. The simulation was computationally intensive, with each run taking approximately 8 h to process.

**Fig. 6:**
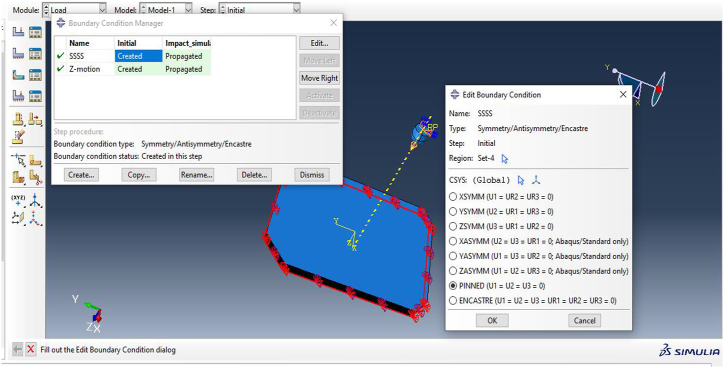
Boundary conditions for the bulletproof vest and bullet motion.

An explicit dynamics analysis was conducted using Abaqus/CAE 2024 to model the interaction between the 7.62 mm projectile and the composite bulletproof vest. Initial conditions for the simulation included specifying the bullet's velocity upon muzzle exit and applying the necessary boundary conditions to the vest. These boundary conditions fixed the back face of the ballistic panel to ensure accurate results during the analysis. The following steps outline the procedure used in the analysis illustrated in Fig. 6.

• Define the initial conditions:

Navigate to **Steps > Initial > Dynamic Explicit** to enter the relevant data for dynamic analysis.

• Assign history output for the simulation:

**Steps > Initial > Predefined Fields**, followed by entering the necessary data to establish the predefined conditions.

The boundary conditions for the vest were applied by fixing the back face and selecting all bottom edges of the polygonal composite plate. These edges were pinned (u1 = u2 = u3 = 0) to restrict movement in all directions. The bullet's velocity was applied to a reference point, directing it towards the vest in a specified direction to simulate the impact event.

**Fig. 7:**
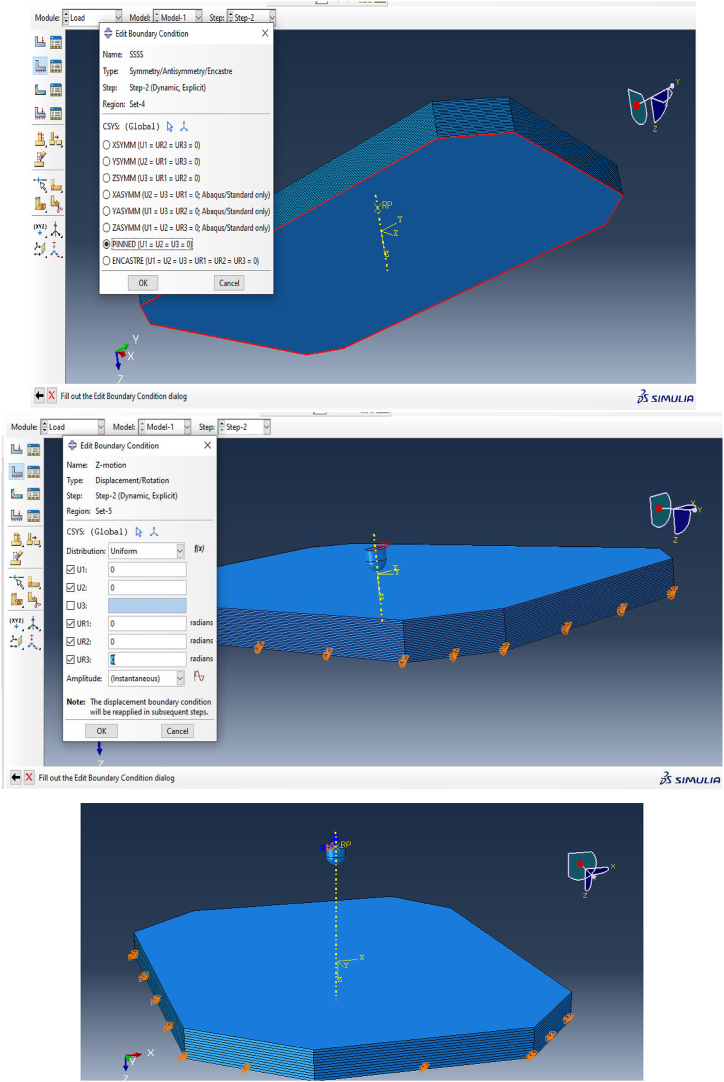
Job creation and monitoring the simulation progress.

**Fig. 8:**
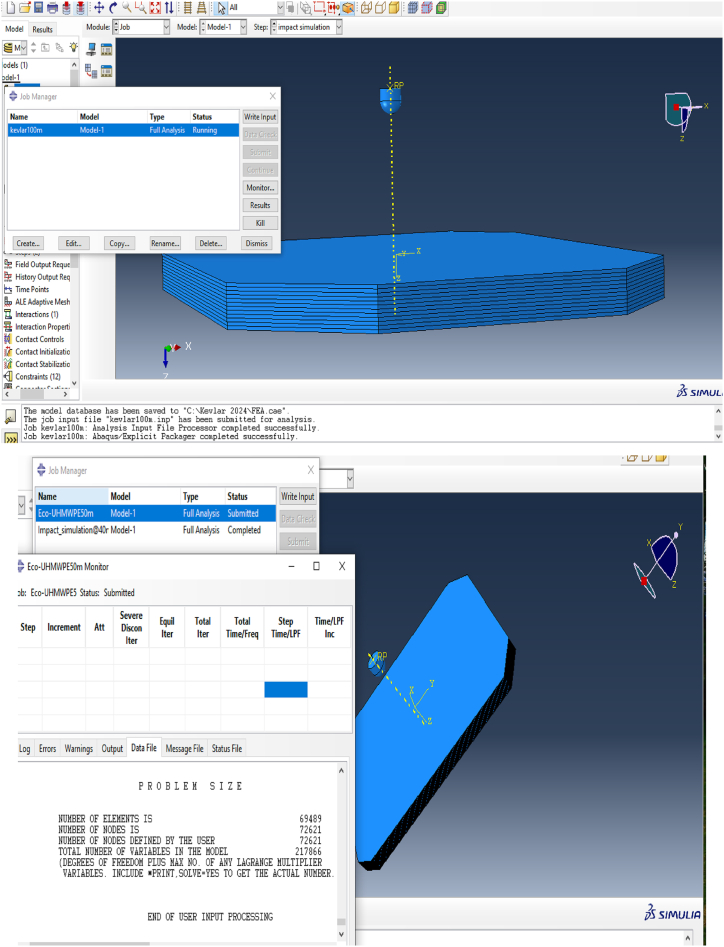
Job creation and monitoring the simulation progress.

After completing the model setup, which included defining the geometry, section properties, contact interactions, and boundary conditions, the simulation was prepared for execution. The Job module in ABAQUS/CAE 2024 was utilized to manage the simulation process effectively. The steps for creating and submitting the job are illustrated in Fig. 7 and Fig. 8.

• **Job module > Create Job > Submit Job** to initiate the simulation.
• Monitor the progress of the simulation in real-time using the job monitoring tools available within Abaqus/CAE 2024.

This process allows for efficient execution and management of the simulation, providing insights into the interaction between the high-velocity projectile and the composite armor.

ECO-UHMWPE (Ultra-High Molecular Weight Polyethylene) exhibits exceptional impact resistance, significantly enhancing its anti-penetration capabilities. Due to its high tensile strength and ability to undergo substantial deformation without fracturing, ECO-UHMWPE efficiently absorbs and dissipates the kinetic energy from ballistic impacts. Numerical simulations demonstrate that ECO-UHMWPE effectively distributes the impact-induced stresses across a larger surface area, reducing the likelihood of full penetration. This superior energy absorption property plays a crucial role in preventing projectile penetration, thereby enhancing the overall protective performance of the armor.

When compared to Kevlar epoxy, which is known for its high tensile strength, ECO-UHMWPE offers several advantages. Despite having a lower tensile strength than Kevlar epoxy, ECO-UHMWPE's lower density and higher elongation result in a more favorable weight-to-protection ratio. This enables the design of lighter ballistic vests without compromising on protective capabilities. The material's ability to maintain its structural integrity under high-velocity impacts further underscores its superior performance. ECO-UHMWPE's high energy absorption efficiency and resilience make it an excellent candidate for modern lightweight body armor, offering reliable protection against high-velocity projectiles.

In conclusion, the ECO-UHMWPE-based bulletproof vest demonstrates outstanding anti-penetration performance, driven by its unique material properties and structural configuration. Its capacity to absorb and dissipate the energy from high-velocity impacts ensures robust protection against ballistic threats, positioning it as a highly viable material for next-generation protective equipment. Numerical analyses further validate ECO-UHMWPE's effectiveness in resisting bullet penetration, confirming its suitability for advanced ballistic applications.

## 3 Result and discussion

This section discusses effects of impact loads on bulletproof vests by analyzing the kinetic energy transfer, stress distribution, and material response to projectile impacts. Specifically, it examines the elastic strain and pressure generated within the vest layers upon impact. A comparative analysis is performed using Abaqus/CAE 2024 to evaluate the deformation, stress distribution, and impact behavior of Kevlar-epoxy and Recycling-UHMWPE vests subjected to projectile impacts at varying distances (ranging from 50 m to 200 m).

The study highlights a significant reduction in vest weight, with the newly designed ECO-UHMWPE vest demonstrating a 22.44 % decrease in mass compared to traditional vests. This weight reduction is achieved without compromising ballistic protection, offering enhanced mobility and comfort for the wearer. The simulations emphasize the benefits of ECO-UHMWPE's lower density while maintaining comparable or superior performance in absorbing impact energy.

Additionally, an analytical approach is employed to assess the failure mode of the ECO-UHMWPE vest. This analysis predicts the likelihood of failure in individual laminate layers when subjected to maximum stress conditions experienced during impact. The insights gained from this study not only confirm the weight-saving advantages of ECO-UHMWPE but also its effectiveness in mitigating ballistic threats, making it a viable material for next-generation body armor.

### 3.1 Finite element analysis (FEA) results for Kevlar-epoxy bulletproof vest

The ballistic performance of the Kevlar-epoxy bulletproof vest was evaluated using finite element analysis (FEA) in Abaqus/CAE 2024. The simulation results provide critical insights into how the vest behaves under high-velocity projectile impact, specifically addressing the stress distribution, strain, pressure, and shear forces experienced by the material.

**Fig. 9:**
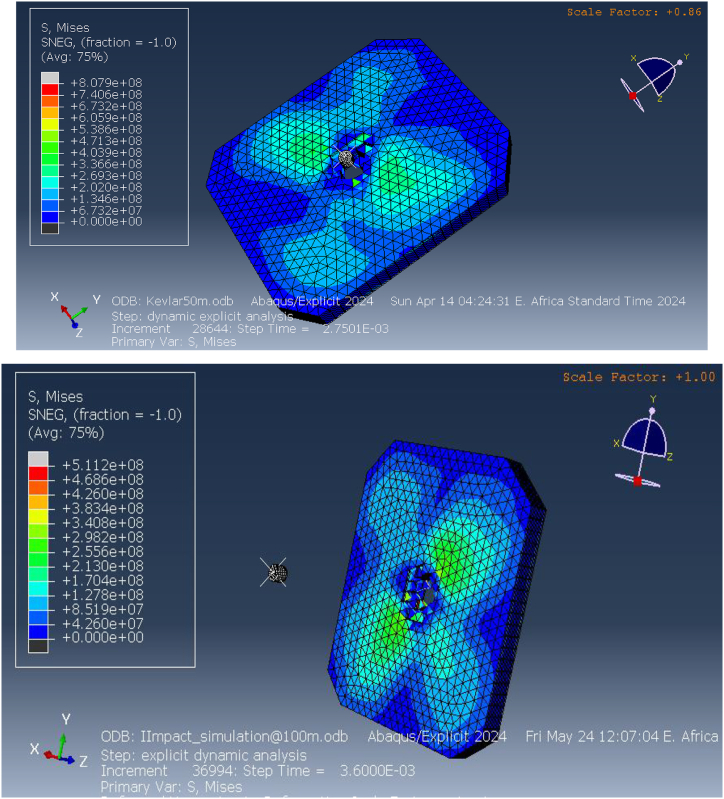
Equivalent (von miss) stress for Kevlar-epoxy bulletproof vest at50m and100m.

One of the key parameters analyzed in the simulation was the equivalent (von Mises) stress, which is a measure of the internal stress response of the material under impact. The results revealed that the equivalent stress values at the impact point reached 807.9 MPa at a distance of 50 m and 511.2 MPa at 100 m, as shown in Fig. 9. These high stress values demonstrate the significant internal forces generated upon impact, indicating that the material near the point of impact experiences intense deformation. The concentrated stress at the impact site suggests that the Kevlar-epoxy material is not fully capable of distributing the load efficiently across the vest. This can lead to localized material failure, particularly in areas directly beneath the projectile. As a result, the vest's capacity to provide adequate protection at closer ranges is compromised, highlighting the limitations of Kevlar-epoxy in high-threat environments.

**Fig. 10:**
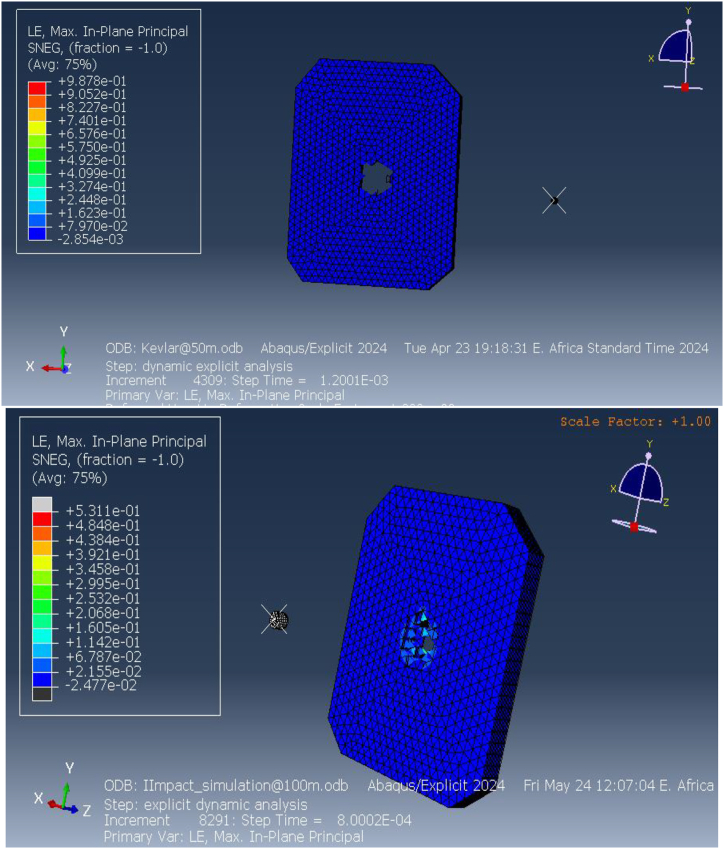
Elastic strain on the Kevlar-epoxy bulletproof vest at 50 m and 100 m.

Further analysis of the elastic strain distribution within the vest, shown in Fig. 10, indicates substantial deformation in areas surrounding the impact point. Elastic strain measures the extent to which the material can deform under stress before returning to its original shape. The simulations revealed that at distances of 50 m and 100 m, the Kevlar-epoxy vest experienced significant reversible deformation. However, at closer ranges (50 m), the strain patterns suggest full perforation of the material, as indicated by the strain values exceeding the material's elastic limit. This perforation is a clear indication of the vest's inability to withstand high-velocity impacts at closer ranges, leading to potential failure. At 100 m, while the deformation was less severe, the strain values still pointed to substantial material stress, indicating the material's limited capacity to absorb energy without undergoing irreversible damage. These findings underscore the importance of improving material resilience, especially for protection against high-velocity projectiles at shorter distances.

**Fig. 11:**
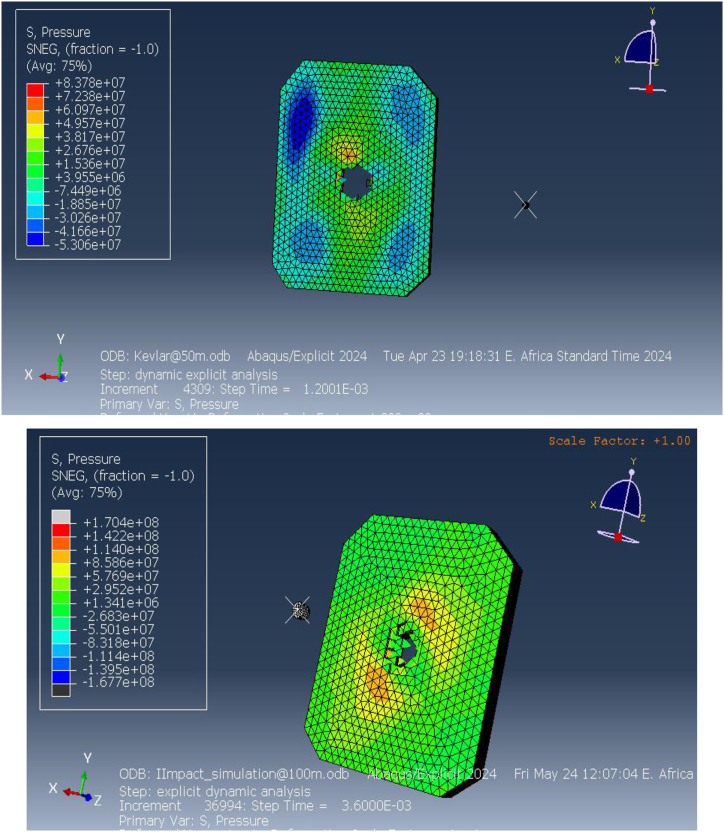
Pressure on the Kevlar-epoxy bulletproof vest at 100 m.

The pressure distribution within the vest, depicted in Fig. 11, further confirms the vest's vulnerability to ballistic impact. The pressure reached a maximum of 83.78 MPa at 50 m and 26 MPa at 100 m, with the highest pressures localized at the center of the impact site. This localization of pressure highlights the vest's inability to effectively spread the impact force over a broader area. As a result, the energy from the projectile is concentrated in a small region, increasing the likelihood of material failure in that area. The significant pressure values at the impact point indicate that the vest's load-bearing capacity is insufficient to prevent high-pressure concentration, particularly at shorter distances. This limitation is critical, as excessive pressure in a localized area can lead to catastrophic failure, leaving the wearer vulnerable to injury.

**Fig. 12:**
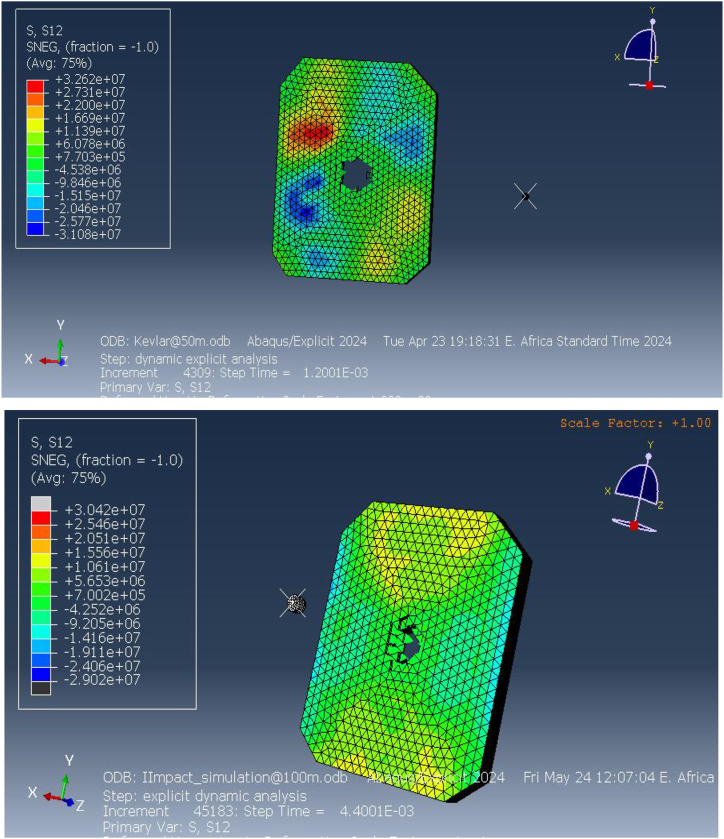
Shear stress on the Kevlar-epoxy bulletproof vest at 50mand100m.

The simulation also analyzed the shear stress distribution, which provides insight into the lateral forces acting on the vest during impact. Fig. 12 shows that the maximum shear stress reached 32.62 MPa at 50 m and 24.2 MPa at 100 m. Shear stress measures the material's resistance to sliding forces, which occur as the projectile interacts with the fibers of the vest. The high shear stress values near the impact point indicate that the Kevlar-epoxy material struggles to resist these forces, particularly at closer distances where the projectile exerts greater force. The concentration of shear stress in specific regions of the vest suggests that the material may fail under these dynamic conditions, further compromising its protective capabilities.

Taken together, the results of the FEA simulations for the Kevlar-epoxy bulletproof vest provide a comprehensive understanding of its mechanical behavior under ballistic impact. The concentration of high equivalent stress, significant elastic strain, localized pressure, and elevated shear stress all point to the material's limitations in effectively absorbing and dissipating the energy from high-velocity projectiles. While the vest demonstrates some capacity for energy absorption, particularly at longer distances (100 m), the results indicate that it is prone to failure under more intense impacts at shorter ranges. This failure is primarily due to the material's inability to spread the impact force across a larger area, resulting in localized stress and strain that exceed the material's tolerance.

The findings suggest that the current Kevlar-epoxy design may not be sufficient to provide adequate protection against high-velocity ballistic threats, particularly at closer ranges. To address these limitations, future research should explore material improvements, such as incorporating more advanced composites or hybrid materials that offer better energy dissipation and stress distribution capabilities. Design modifications that enhance the structural integrity of the vest, such as optimizing the layering configuration or integrating additional protective layers, could also improve its performance. These improvements would be essential in ensuring that the vest can provide reliable protection in a wider range of ballistic threat scenarios, ultimately enhancing wearer safety in high-risk environments.

### 3.2 Deformation analysis of a Kevlar-epoxy composite bulletproof vest

**Fig. 13:**
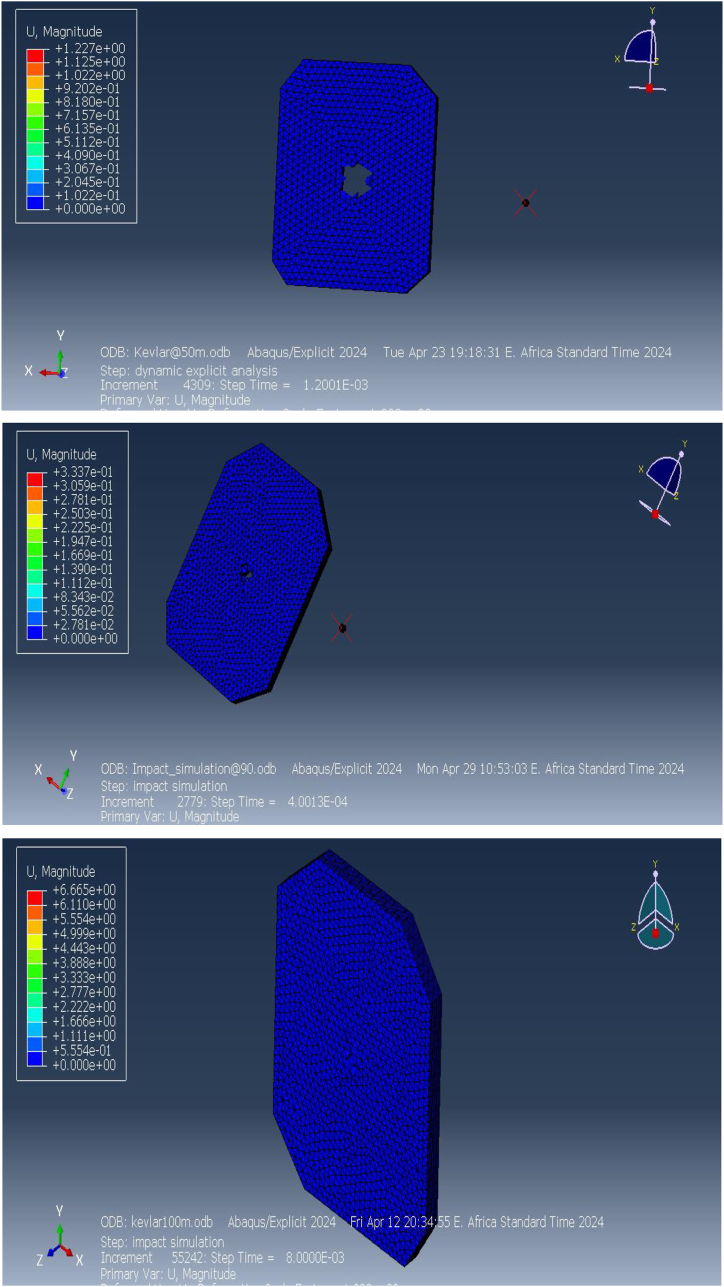
The deformation of Kevlar-epoxy vest at 50m, 90m, and 100 m.

The deformation response of the Kevlar-epoxy composite bulletproof vest was simulated under different impact distances to assess its protective performance. Fig. 13 presents the deformation profiles at distances of 50 m, 90 m, and 100 m. The simulation results clearly indicate that the projectile successfully penetrates the vest at all three distances. At 50 m, the vest experiences substantial deformation concentrated at the impact site, leading to full perforation and implying a significant risk of injury to the wearer. This deformation suggests that at such close ranges, the Kevlar-epoxy composite is incapable of absorbing the high kinetic energy imparted by the projectile, resulting in a failure to prevent penetration.

At 90 m, while there is a slight reduction in deformation compared to 50 m, the projectile still manages to penetrate the vest, indicating that the vest does not provide sufficient resistance at this distance either. Similarly, at 100 m, the deformation pattern shows localized material displacement at the impact site, but the vest continues to exhibit insufficient energy absorption, allowing the projectile to perforate it completely. These results highlight the limitations of the Kevlar-epoxy composite vest in ballistic protection at the distances tested, particularly under high-velocity impacts, where energy absorption and dissipation mechanisms are overwhelmed.

Bulletproof vests are designed to absorb and distribute the energy of an impacting projectile to prevent penetration and reduce the risk of injury. However, the results from these simulations indicate that the Kevlar-epoxy composite fails to meet these objectives under the tested conditions, leading to perforation and compromised protection.

**Fig. 14:**
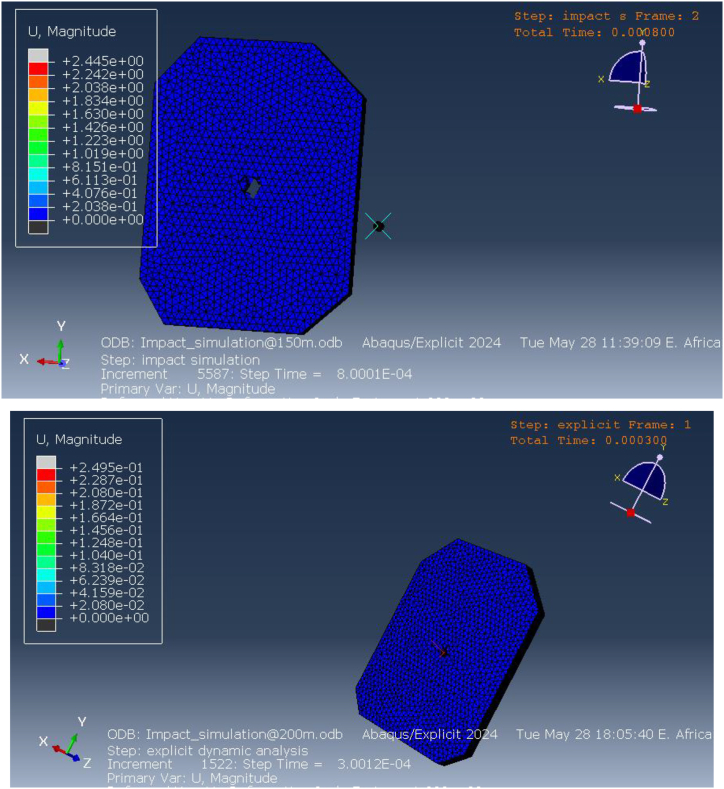
Deformation of Kevlar-epoxy vests at 150mand200m range.

Further simulations were conducted at longer distances of 150 m and 200 m to explore the vest's deformation behavior at reduced projectile velocities. Fig. 14 shows the deformation results at these extended ranges. At 150 m, the maximum deformation is measured at approximately 2.8 mm, concentrated at the impact point. While this value indicates that the vest absorbed a portion of the projectile's kinetic energy, the significant deformation suggests that the vest's integrity is compromised, raising concerns about the potential for penetration or injury. Although full perforation was not observed, the vest's performance at this range reveals that it is still at risk of failure due to the localized concentration of deformation.

At 200 m, the maximum deformation decreases to about 2.2 mm, which corresponds to the lower kinetic energy of the projectile at this longer distance. The reduced deformation pattern at 200 m suggests improved energy absorption and dissipation by the vest, with less concentrated stress at the impact point. However, the deformation remains focused around the impact site, and while perforation was avoided, the significant displacement of material still poses a risk to the wearer, particularly in scenarios involving multiple impacts or higher-energy projectiles.

These deformation patterns provide important insights into the behavior of the Kevlar-epoxy vest under dynamic impact conditions. At shorter distances (50 m, 90 m, and 100 m), the vest fails to stop the projectile, indicating a clear need for material improvements or design optimization. Even at longer distances (150 m and 200 m), where projectile velocity is lower, the deformation is significant enough to question the vest's overall protective effectiveness. Although perforation does not occur at these longer distances, the magnitude of deformation suggests that the wearer could still be at risk from internal injury due to the vest's inability to fully dissipate the impact energy.

In summary, the deformation analysis of the Kevlar-epoxy bulletproof vest demonstrates the limitations of the material in resisting high-velocity projectile impacts. The vest's inability to prevent penetration at shorter distances and its substantial deformation at longer distances underscore the need for further research into alternative materials and design modifications that could enhance its ballistic resistance. The results emphasize that while Kevlar-epoxy composites offer some degree of protection, they may not be sufficient in high-stress scenarios, necessitating the development of more robust solutions for ensuring wearer safety.

### 3.3 Results of the FEA for ECO-UHMWPE bulletproof vest

This section presents the finite element analysis (FEA) results for the ECO-UHMWPE bulletproof vest under ballistic impacts at different ranges. The analysis examines key parameters, including Equivalent (von Mises) stress, directional stresses along the x and y axes, total deformation, elastic strain, and pressure distribution. These simulations, conducted at 50 m and 100 m ranges, provide crucial insights into the mechanical performance of the ECO-UHMWPE vest when subjected to high-velocity projectile impacts.

The simulation results reveal that the highest Equivalent (von Mises) stress values at 50 m and 100 m are +41.40 GPa and +28.76 GPa, respectively, with the stress concentrations represented in red in the stress maps. This high stress at shorter distances indicates a significant load-bearing capacity of the material, especially at 50 m where the impact velocity is higher. The von Mises stress provides a measure of the material's distortion energy, and these values reflect the extreme conditions under which the vest is tested to assess its ballistic performance.

**Fig. 15:**
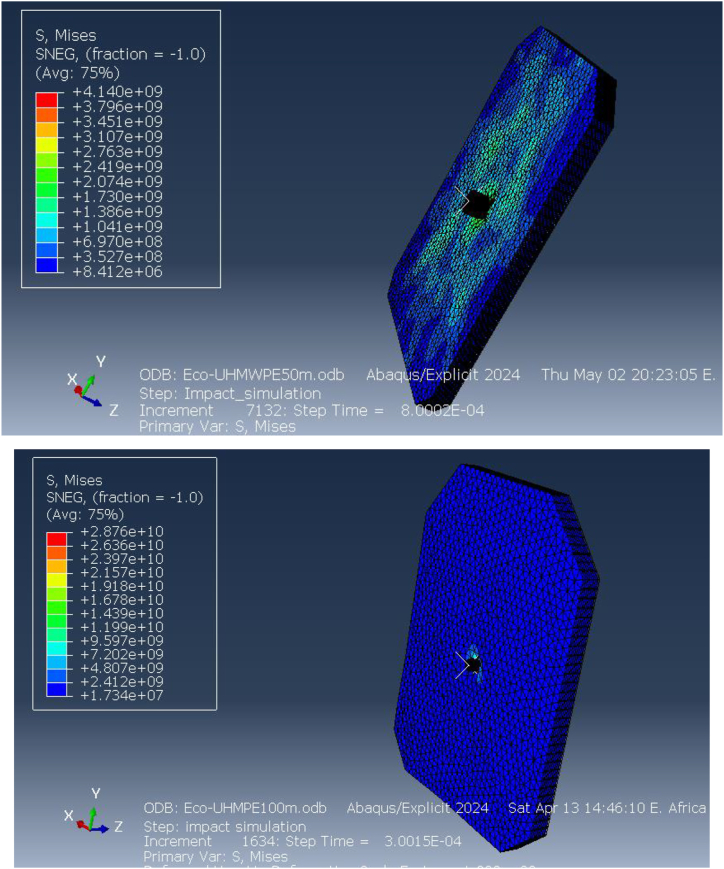
Equivalent (von miss) stress for ECO-UHMWPE vest from 50to100m range.

Fig. 15 illustrates the elastic strain distribution in the ECO-UHMWPE bulletproof vest at impact distances of 50 m and 100 m. At 50 m, the elastic strain reaches a maximum of 6.906e-2, indicating substantial deformation due to the high projectile velocity and energy. This high strain value suggests that the material is experiencing significant reversible deformation but is still maintaining structural integrity. In contrast, at 100 m, the elastic strain drops to 3.530e-1, reflecting reduced deformation due to the lower kinetic energy of the projectile at this range. The reduction in strain with increased distance demonstrates the vest's capacity to adapt to different threat levels by mitigating deformation at lower impact velocities.

**Fig. 16:**
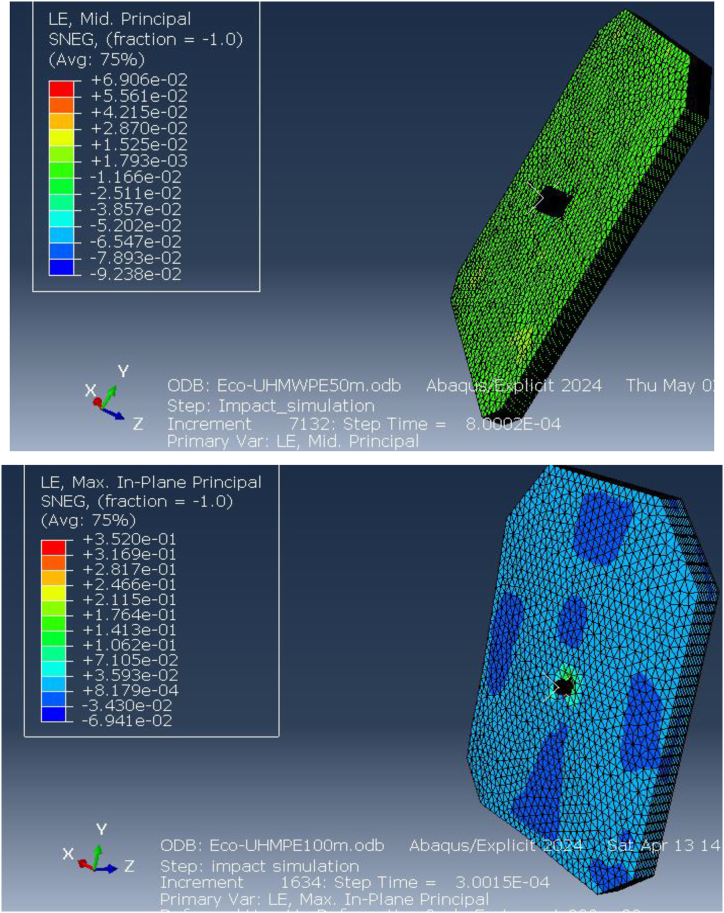
Elastic strains on the ECO-UHMWPE vest from 50m to 100m range.

Fig. 16 further highlights the elastic strain distribution across the vest, emphasizing the material's ability to absorb and dissipate energy under ballistic impact. The vest's performance under varying strain conditions is crucial for optimizing its design for different threat levels.

**Fig. 17:**
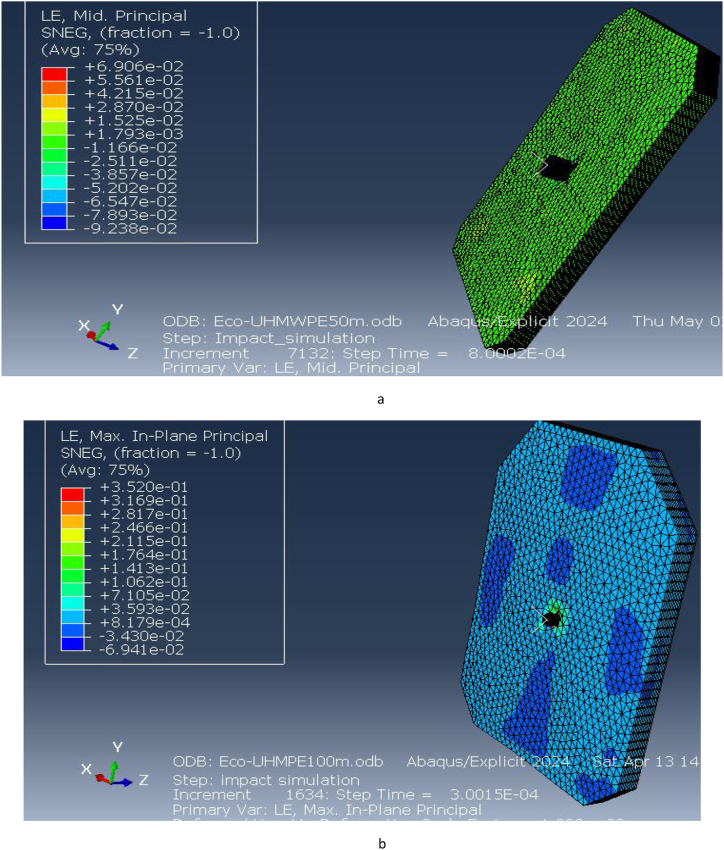
[a, b] Distribution Pressure on ECO-UHMWPE vest at 40m and 50m range.

The pressure distribution across the ECO-UHMWPE vest is presented in Fig. 17(a and b). At a range of 40 m, the applied pressure on the vest is approximately 1.124e9 Pa, while at 50 m, the pressure decreases slightly to 1.098e9 Pa. This high-pressure concentration around the impact point suggests that the vest effectively absorbs and redistributes the energy imparted by the projectile, preventing penetration and protecting the wearer from severe injury. The pressure distribution serves as a key indicator of the vest's ability to withstand external forces and dissipate energy efficiently.

**Fig. 18:**
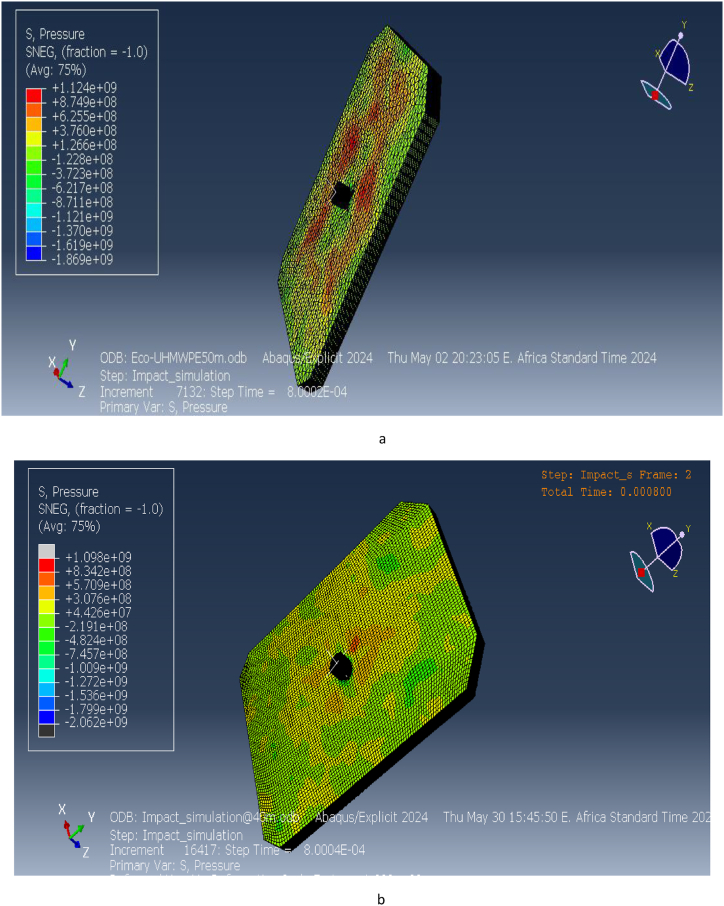
[a, b] Stress on x-direction of ECO-UHMWPE at 50mand100m range.

**Fig. 19:**
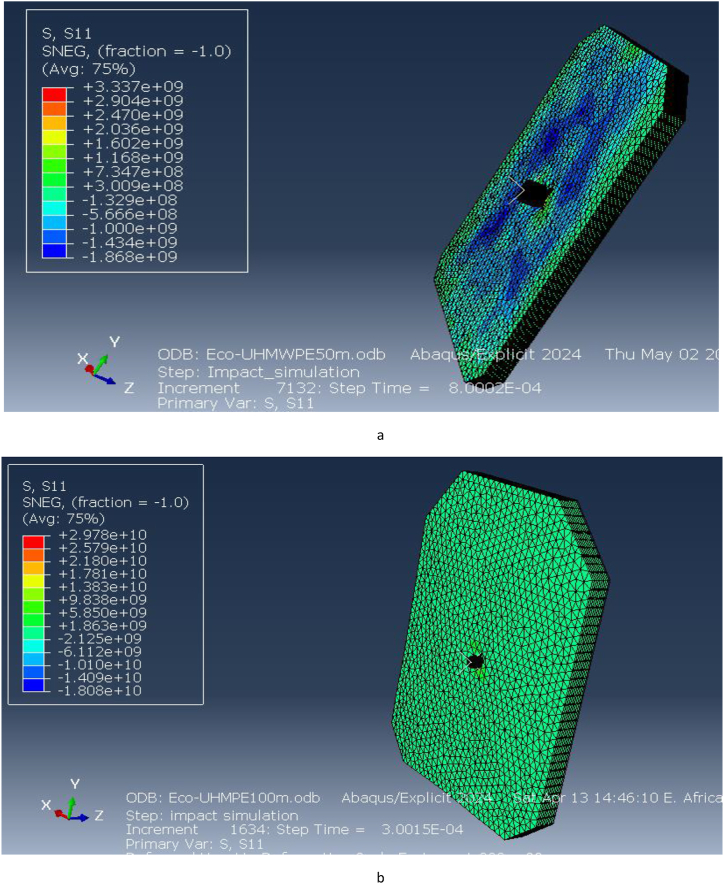
Stress on y-direction of ECO-UHMWPE from 50m to 100m range.

Directional stresses along the x and y axes were evaluated, as illustrated in Fig. 18(a and b) and Fig. 19(a and b). At 50 m, the stress along the x-direction is 3.337e9 Pa, and at 100 m, it is 2.978e10 Pa. Similarly, the stress along the y-direction at 50 m is 2.474e9 Pa, while at 100 m, it increases to 5.598e9 Pa. These results indicate that the vest experiences significant stress in both directions under impact, with higher stresses at shorter distances due to the increased impact velocity. The ECO-UHMWPE material's high tensile strength and energy absorption capacity allow it to withstand these stresses, preventing catastrophic failure at critical impact points.

The stress applied on the x direction of the bulletproof vest is at 50m 3.337e9and at 100m is 2.978e10.

**Fig. 20:**
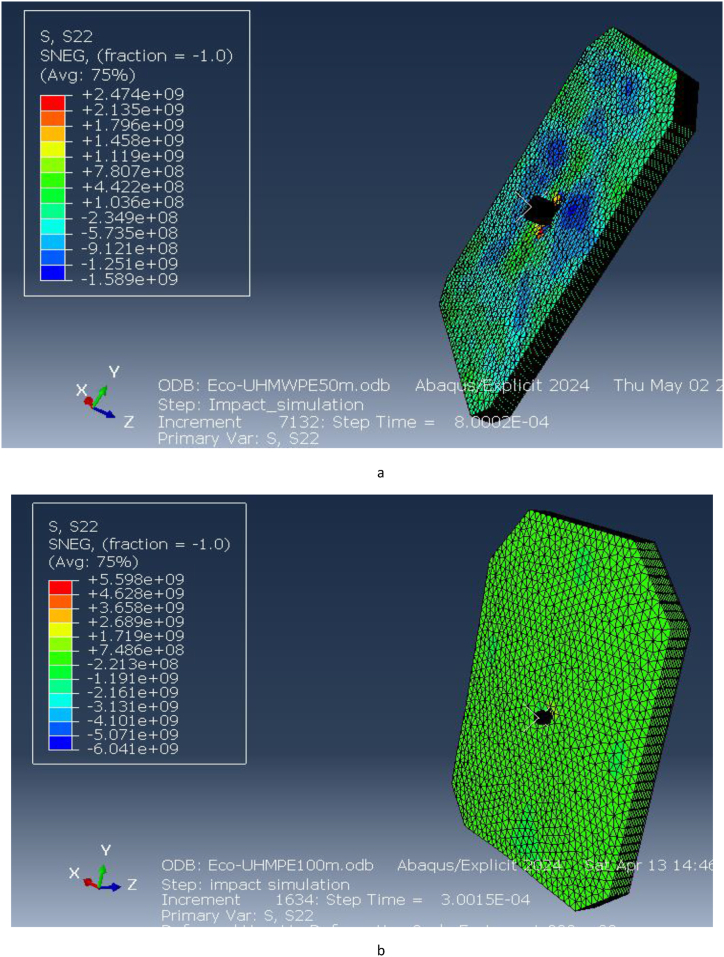
[a, b] Shear stress on the ECO-UHMWPE vest at 50m and100m ranget.

Fig. 20 (a, b) illustrates the shear stress distribution on the ECO-UHMWPE vest. At a distance of 50 m, the simulation shows a pronounced concentration of shear stress near the impact point, reflecting the material's ability to absorb and dissipate energy under high-velocity impacts. As the range increases to 100 m, the shear stress concentration diminishes, indicating reduced energy absorption due to the projectile's lower velocity. These results emphasize the material's effectiveness in mitigating close-range threats while highlighting its potential limitations in maintaining stopping power at extended distances.

Overall, the FEA results demonstrate that ECO-UHMWPE is highly effective in absorbing and redistributing the forces generated by ballistic impacts. Its superior tensile strength, low density, and excellent energy absorption properties make it an ideal candidate for ballistic protection, especially at close ranges. However, the reduction in stress concentration and shear stress at longer distances suggests that further material optimization may be necessary to enhance its stopping power for more distant threats.

### 3.4 Deformation analysis of ECO-UHMWPE bulletproof vest

**Fig. 21:**
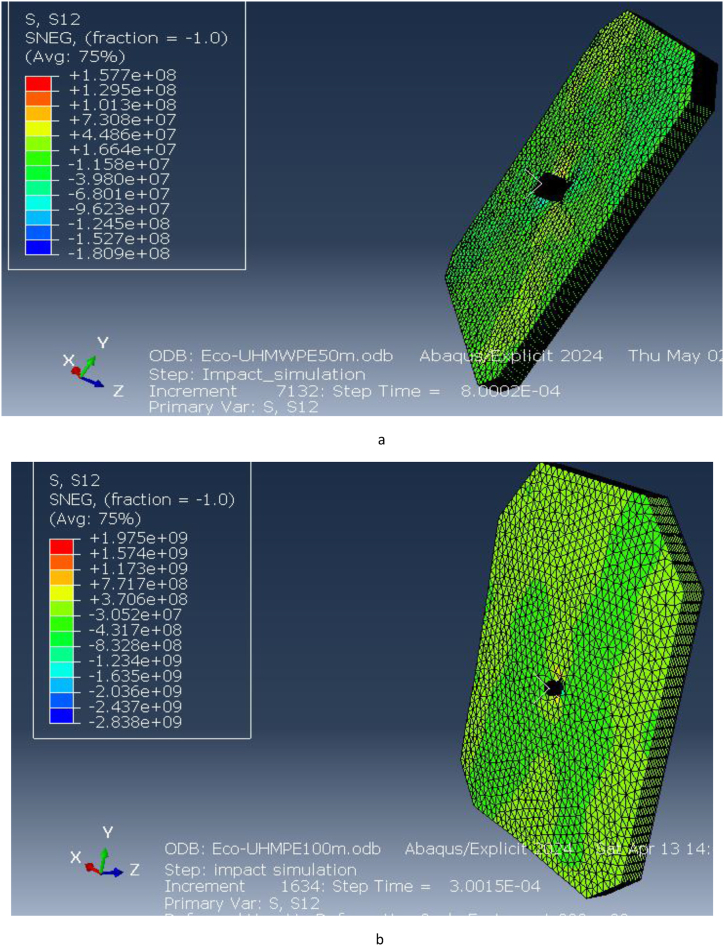
Stress on y-direction of ECO-UHMWPE from 50m to 100m range.

The deformation behavior of the ECO-UHMWPE bulletproof vest plays a vital role in assessing its protective performance. Fig. 21(a and b) depicts the deformation profiles of the vest under ballistic impacts at distances of 40 m, 50 m, and 100 m. The simulations, conducted using ABAQUS/CAE 2024, were aimed at evaluating the material's response to varying impact velocities and understanding how it deforms under different ballistic conditions.

**Fig. 22:**
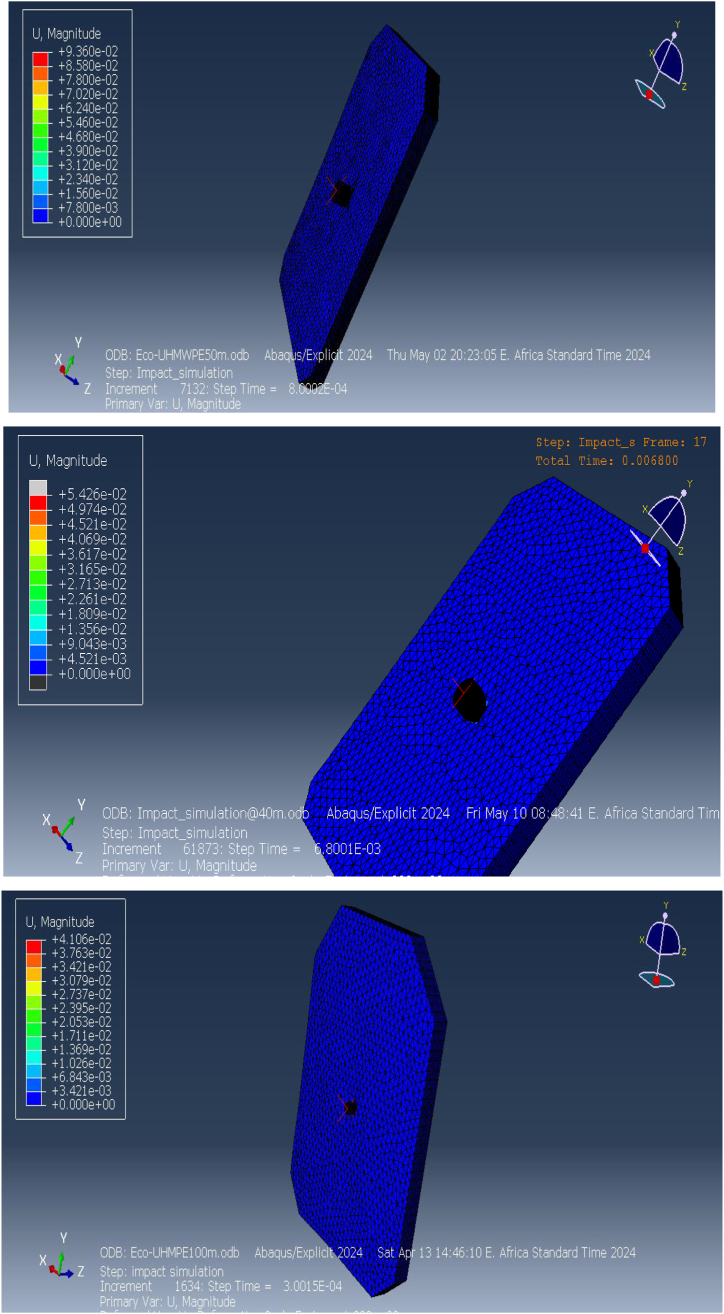
Analysis of ECO-UHMWPE Vest Deformation at 40m, 50m, and100m.

At a distance of 40 m, the ECO-UHMWPE vest demonstrates a maximum deformation of approximately 9. mm, concentrated at the point of impact. This outward bulge occurs as the vest absorbs the kinetic energy of the projectile, resulting in plastic deformation of the material. The extent of deformation reflects the vest's capacity to dissipate a significant portion of the projectile's energy, effectively preventing penetration. Despite the substantial deformation, the vest retains its structural integrity, offering critical protection to the wearer by distributing the impact energy over a broader area, as illustrated in Fig. 22.

At 50 m, the deformation remains similar to that observed at 40 m, indicating that the ECO-UHMWPE material continues to perform reliably under high-energy impacts. As the distance increases to 100 m, the deformation reduces to approximately 6.8 mm due to the decreased impact velocity of the projectile at longer ranges. While the deformation is less pronounced at this range, the vest still effectively absorbs the projectile's residual energy, showcasing its capability to provide robust ballistic protection even at extended distances. The analysis, depicted in Fig. 22, highlights the material's effectiveness in maintaining protective performance across varying impact ranges.

The deformation analysis offers quantitative evidence of the vest's energy absorption efficiency. The observed deformation values at different ranges underscore the robustness of ECO-UHMWPE in withstanding high-velocity impacts while minimizing the risk of penetration and injury to the wearer. By redistributing and absorbing impact energy, this material demonstrates significant potential for use in next-generation bulletproof vests.

### 3.5 Performance Analysis of ECO-UHMWPE bulletproof vest: strain evaluation

**Fig. 23:**
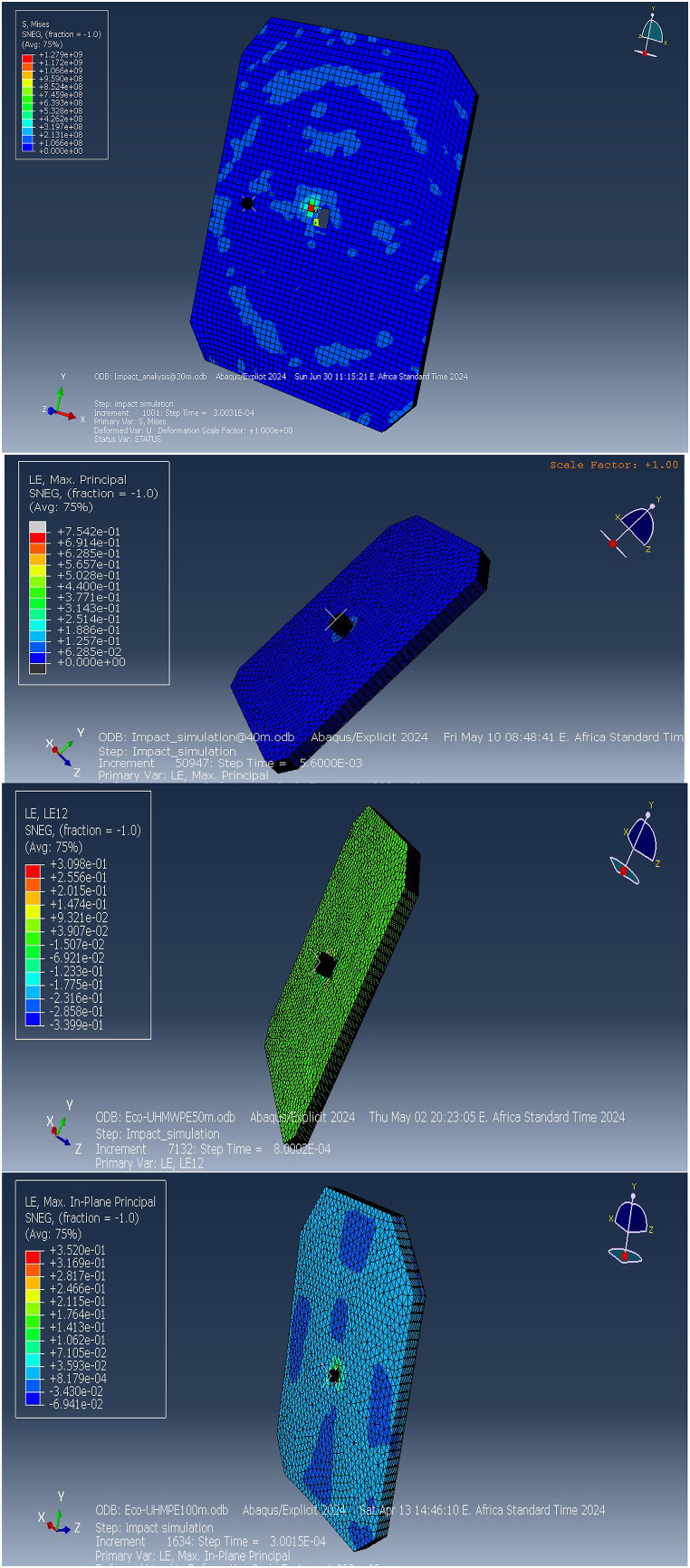
Strain Energy and Performance Analysis of ECO-UHMWPE Vest at Impact Distances of 30 m, 40 m, 50 m, and 100 m.

A comprehensive performance evaluation of the ECO-UHMWPE (Ultra-High-Molecular-Weight Polyethylene) bulletproof vest was conducted using simulated strain data from ballistic impacts at various distances, including 30 m, 40 m, 50 m, and 100 m, as illustrated in Fig. 23. The highest elastic strain was observed at 30 m, measuring 9. mm, and progressively declined with increasing distance, with values of 6.52 mm at 50 m and 4.89 mm at 100 m. This reduction in strain corresponds to the material's decreasing energy absorption as the projectile velocity diminishes over longer ranges. The concept of strain energy, which represents the material's ability to absorb energy during deformation, is fundamental to understanding this behavior. In the context of ballistic protection, greater strain energy indicates an improved capacity to absorb and dissipate impact energy, thus enhancing the vest's protective performance.

While the reduced strain at 100 m may suggest lower deformation, it could also imply less efficient energy absorption by the vest. On the other hand, the elevated strain observed at 40 m indicates a higher degree of deformation, which suggests greater strain energy absorption. This enhanced energy dissipation at closer ranges, particularly at 40 m, suggests that the vest may offer superior protection by effectively managing the bullet's kinetic energy through controlled deformation.

At extended ranges of 150 m and 200 m, the ECO-UHMWPE vest maintained its anti-penetration integrity, exhibiting lower stress values and minimal deformation. These results highlight the vest's ability to provide reliable ballistic protection even at varying impact velocities. The high tensile strength, substantial elongation capacity, and low density of ECO-UHMWPE are key material properties that contribute to its exceptional performance in protective applications.

Numerical simulations and analyses confirm the vest's efficacy in preventing bullet penetration across different operational conditions. However, it is important to note that at 30 m, the vest was fully perforated, indicating a failure to provide adequate protection at this range.

### 3.6 Equivalent (von miss) stress for Recycling-UHMWPE bulletproof vest

**Fig. 24:**
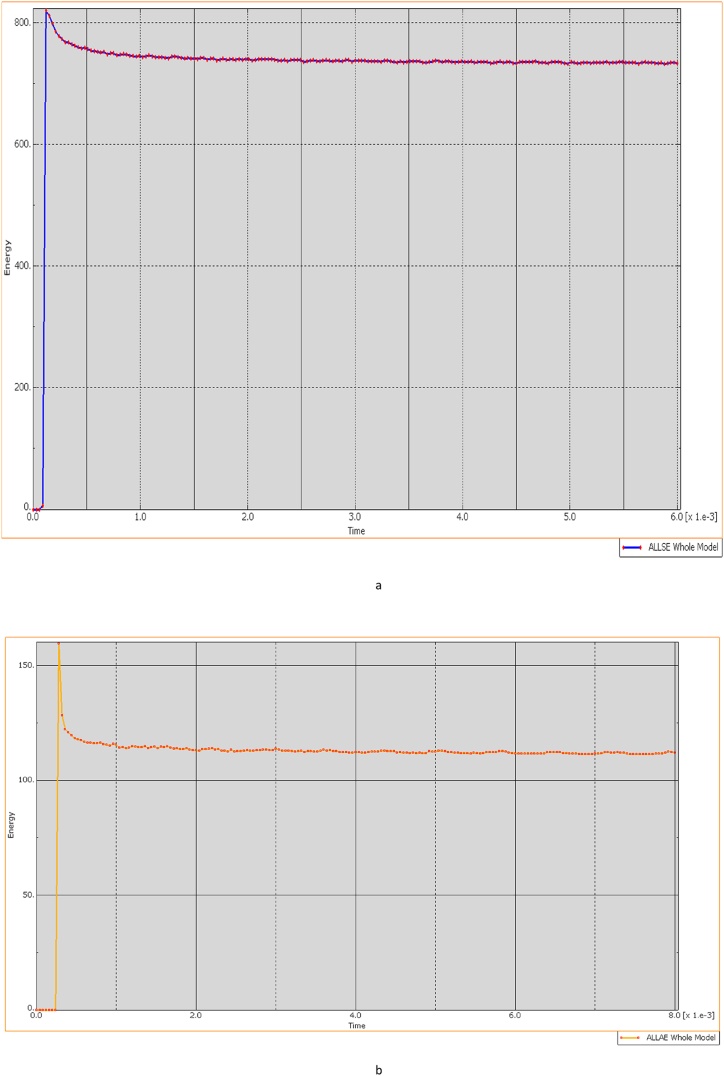
Total energy Damage dissipation for Eco-vest and Kevlar.

**Fig. 25:**
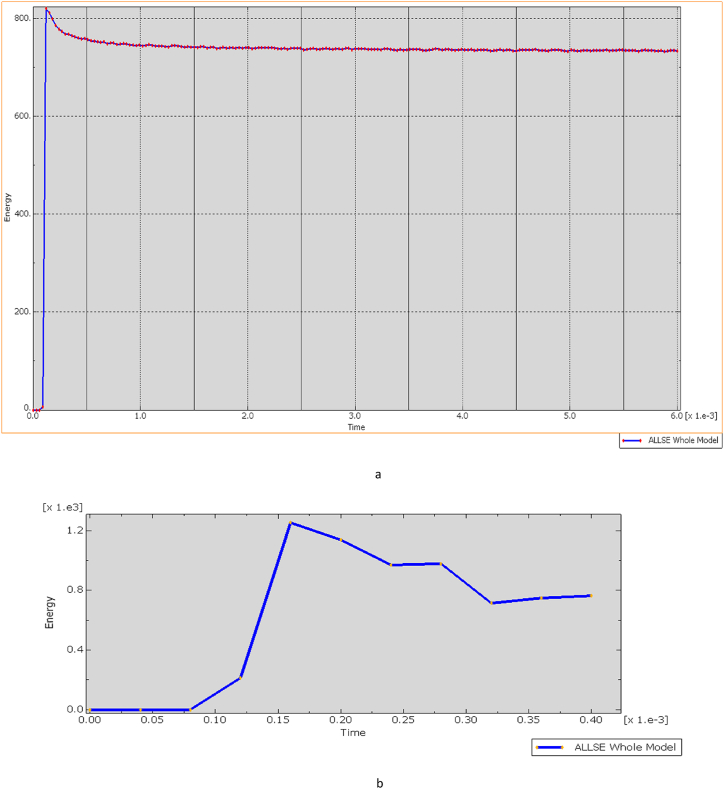
[a, b] Elastic strain energy Vs time increment for Eco-UHMWPE, and Kevlar.

Fig. 24(A, B) illustrates that the Von Mises stress induced in the bulletproof vest at an impact velocity of 838 m/s from a distance of 50 m reaches 2.9 GPa. This stress level is below the composite material's maximum tensile strength of 3.6 GPa and its compression limits, indicating that the vest can effectively withstand the ballistic impact without sustaining structural damage. Consequently, the ECO-UHMWPE material demonstrates sufficient resilience to absorb and dissipate the impact energy, ensuring enhanced protection under high-velocity conditions (see Fig. 25).

**Fig. 26:**
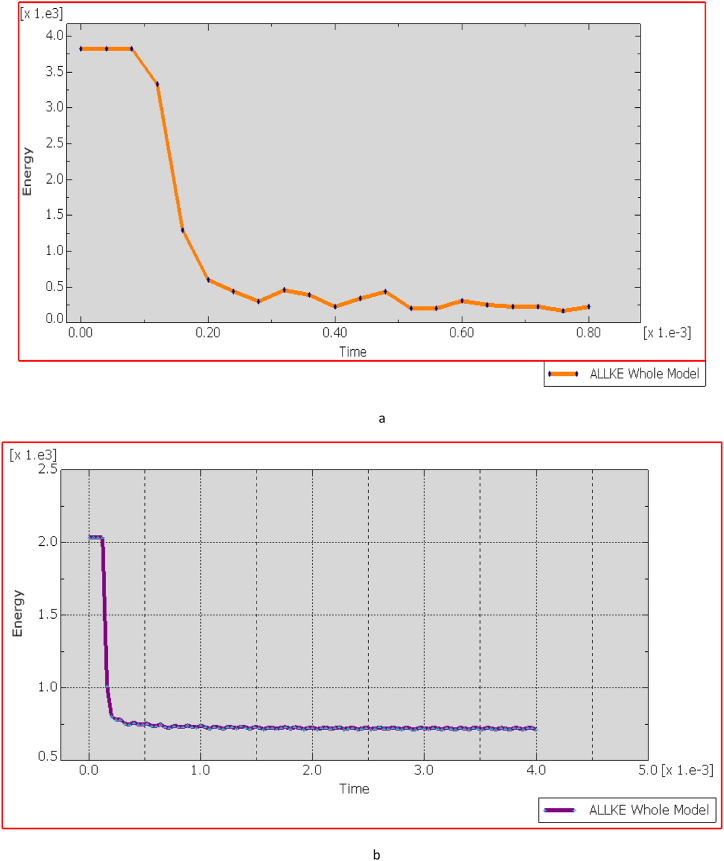
(a, b) Kinetic energy of the projectile on both deformed body armor.

Fig. 25 (A, B) shows the elastic strain distribution in the Kevlar vest, revealing significant strain at shorter impact distances, which indicates a high deformation level and a nearing of its elastic limit. This elevated strain could lead to structural failure, as the Kevlar may be unable to recover its original form, reducing its effectiveness against further impacts. In contrast, Fig. 26 presents the strain response in the ECO-UHMWPE vest, where notably lower strain values reflect enhanced control over deformation. With a maximum strain of 3.5300e-1 and a minimum of −6.9412e-2 at 838 m/s, the ECO-UHMWPE material demonstrates effective energy absorption and structural integrity retention. This superior performance underscores ECO-UHMWPE's capability to withstand high-velocity impacts while preserving its protective properties, making it a preferable material for improved ballistic resistance.

Fig. 26 (A, B) illustrates the relationship between kinetic energy (KE) in Joules (J) and time (t) in milliseconds (ms), where a bullet with an initial KE of 00 J representative of a high-powered rifle round approaches a bulletproof vest at 50 m. The graph demonstrates that KE remains stable at 00 J until impact (around 0.1 ms), reflecting the bullet's consistent velocity prior to contact. Upon impact, a rapid drop in KE occurs, decreasing sharply from 00 J to 500 J within just 0.02 ms, signifying a substantial transfer of energy (approximately 80 %) from the bullet to the vest. This indicates that the vest absorbs the energy effectively, preventing further penetration, as evidenced by the residual 500 J kE, which the vest safely dissipates.

The graph also reveals oscillatory behavior post-impact, indicating the vest material's dynamic response as it absorbs and redistributes energy. These oscillations suggest that the material effectively manages the energy transfer over time, with the initial peak marking the impact moment. Both the Kevlar-epoxy and ECO-UHMWPE vests exhibit this initial peak, demonstrating their capacity to absorb the kinetic energy during impact.

### 3.7 Ballistic performance analysis through strain energy distribution

**Fig. 27:**
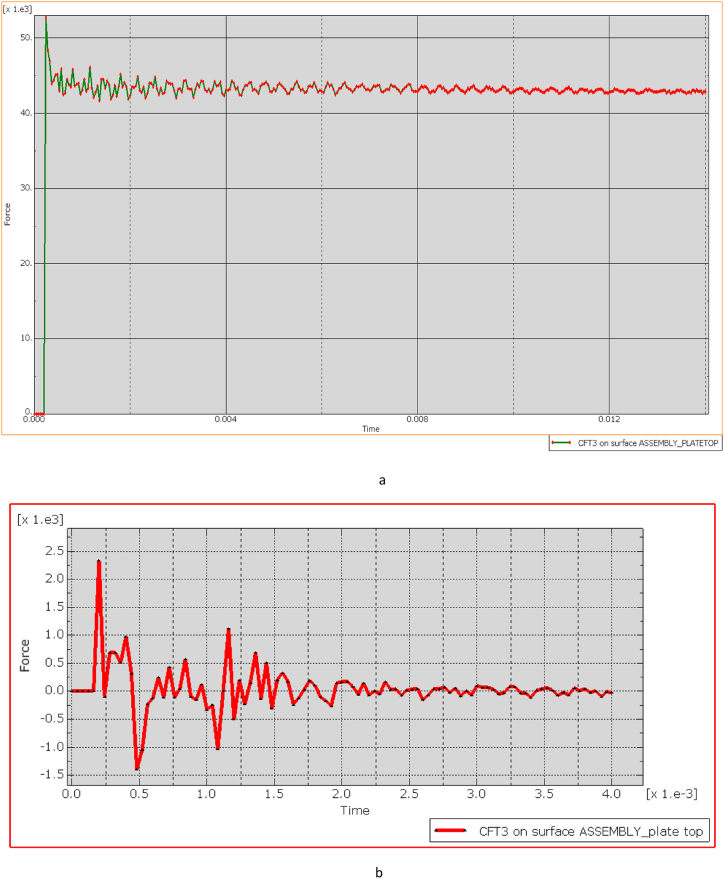
(a, b) Ballistic Performance Analysis Through Strain Energy Distribution.

The ballistic simulations illustrated in Fig. 27 (a, b) offer critical insights into strain energy distribution within a deformed ECO-UHMWPE vest, serving as a valuable reference for assessing its material performance under impact. The graph reveals a progressive increase in strain energy, beginning at 0.1 MJ/m³ at the point of impact and rising to 0.5 MJ/m³ across a 10 mm distance from the impact zone. This distribution suggests that ECO-UHMWPE effectively absorbs and disperses impact energy throughout its volume, potentially enhancing ballistic resistance.

A distinct, sharp peak in strain energy followed by a rapid decline, however, would indicate localized material failure, such as fractures or cracking. Comparative analysis of strain energy distribution for ECO-UHMWPE and other materials, such as Kevlar, under equivalent conditions highlights differences in performance. As shown in Fig. 27 (a), the ECO-UHMWPE vest reaches a maximum strain energy approximately 0.1 μs (μs) post-impact, representing its immediate response to the load. The subsequent exponential decline in strain energy suggests plastic deformation within the ECO-UHMWPE vest as it dissipates impact energy. Conversely, the Kevlar-epoxy vest exhibits a lower threshold for impact resistance against a 7.62 mm projectile, likely attributed to its material properties.

The strain energy graph for ECO-UHMWPE shows substantial fluctuations initially, peaking above 1000 units, before gradually stabilizing with diminishing oscillations. This pattern signifies an effective dynamic response, with high initial energy absorption followed by a sTable redistribution of stress. In contrast, the Kevlar-epoxy graph exhibits an initial rapid increase to approximately 400 units, followed by a swift decline, indicating limited capacity to absorb and retain impact energy. These findings underscore ECO-UHMWPE's superior energy absorption characteristics in comparison to Kevlar-epoxy under identical ballistic conditions.

### 3.8 Deformation and impact: ECO-UHMWPE vs. Kevlar- vests

A comparative analysis of deformation and impact response indicates that ECO-UHMWPE vests exhibit superior performance over Kevlar-epoxy vests, primarily due to lower elastic strain levels. This characteristic minimizes deformation and accelerates energy dissipation, as observed through quicker kinetic energy damping on a time-kinetic energy graph. Consequently, ECO-UHMWPE vests demonstrate enhanced ballistic protection, reflected by the lower residual kinetic energy post-impact in simulation results. The faster stabilization of ECO-UHMWPE further suggests its suitability for repeated impacts, as it effectively dissipates energy while maintaining structural integrity.

**Fig. 28:**
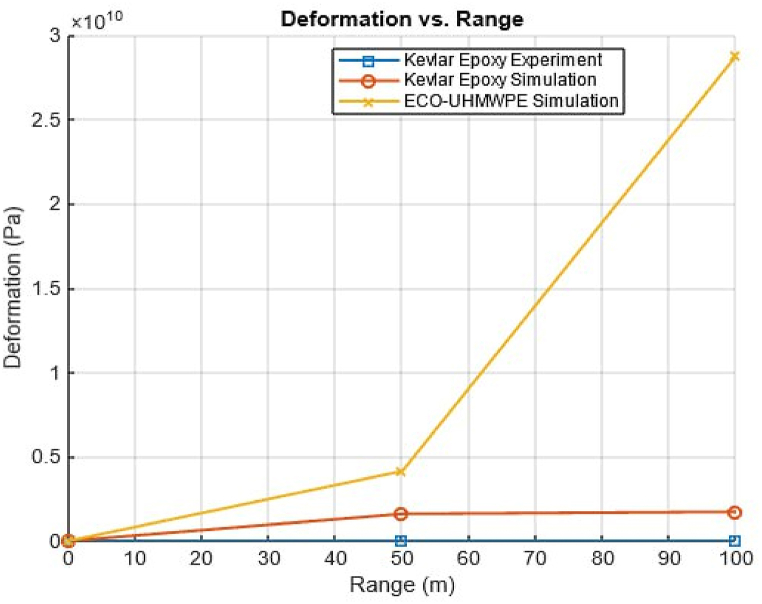
Range of fire vs deformation.

The deformation and penetration effects on ECO-UHMWPE and Kevlar-epoxy vests across various firing ranges are presented in Table 5, illustrating the consistently lower deformation and improved impact resistance of ECO-UHMWPE at equivalent distances. Fig. 28 Visually depicts the relationship between firing range and the resulting deformation for both vest materials.

**Table 5:** Ballistic performance analysis through strain energy distribution.

Range	ECO-UHMWPE	Kevlar-epoxy
30 m	Fully penetration (Perforated)	Perforated
40 m	Penetration exists Deformation is 9.mm	Perforated
50 m	Penetration exists Deformation is 6.520 mm	Perforated
90 m	Penetration exists Deformation is 4.89 mm	Perforated
100 m	Penetration exists Deformation is 4.106 mm	Perforated
150 m	Penetration exists Deformation is 2.8 mm	Penetration exists Deformation is 2.81 mm
200 m	Penetration exists Deformation is 2.2 mm	Penetration exists Deformation is 2.74 mm

### 3.9 Comparison of the kevlar and ECO-UHMWPE vests by FEM

Finite Element Method (FEM) simulations are used to conduct a comparative analysis between Kevlar and ECO-UHMWPE bulletproof vests, focusing on key performance metrics such as deformation, impact resistance, and stress distribution under ballistic impacts. Simulations assess projectile impacts from distances of 50m, 100m, 150m, and 200m. For each 50m increase in range, the projectile's velocity is reduced by approximately 10 % from the initial muzzle velocity of 838 m/s, following the findings by Zou et al.

The FEM results demonstrate that ECO-UHMWPE provides enhanced ballistic protection compared to Kevlar. This material's ability to distribute stress evenly and absorb impact energy minimizes deformation and reduces stress concentration, thus enhancing its reliability as protective equipment.

**Fig. 29:**
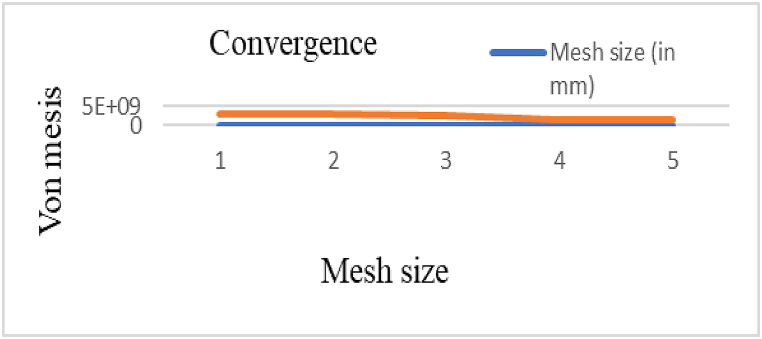
Grid independence test.

The results of the Von Mises stress values at varying distances for both Kevlar and ECO-UHMWPE vests, impacted by projectiles with an initial velocity of 838 m/s, are summarized in Table 6. The grid independence test conducted to ensure the accuracy of the simulations is depicted in Fig. 29.

**Table 6:** Grid independence test.

Mesh type	Mesh size (mm)	Node	Element
Course for vest	0.003	323037	162489
Fine	0.0002	989764	969114

### 3.10 Failure analysis of ECO-UHMWPE fiber

The failure analysis of ECO-UHMWPE (Environmentally Correct Ultra-High-Molecular-Weight Polyethylene) fiber involves evaluating critical parameters such as tensile strength, elongation at break, and energy absorption capacity to understand its performance under stress and impact conditions. Based on simulations, the maximum stress components occurred at a distance of 50 m, where the stress values were determined as σ1 = 09.89 MPa, σ2 = 09.89 MPa, and τ12 = 173.2 MPa. Using the Tsai-Wu failure criterion, which predicts laminate failure based on plane stress and strain relationships, the coefficients H1, H2, H6, H11, H22, H66, and H12 were derived from the strength parameters of a unidirectional lamina (as per Equation (27)). For the ECO-UHMWPE material, the ultimate tensile and compressive strengths in the longitudinal direction are 09.89 MPa, while the ultimate tensile and compressive strengths in the transverse direction are 3000 MPa and 2900 MPa, respectively [28]. Based on the simulation for ECO-UHMWPE the component of the stress has a maximum result at 50m so, by using Tsai Wu failure criteria [2]: Based on Tsai-Wu in 1976 applied the failure theory that the proposes for the metal and composite lamina to find plane stress and strain to analyze laminate failure.

A lamina will be failed if;

(27) $$H_{1}\sigma_{1}+H_{2}\sigma_{2}+H_{6}\tau_{12}+H_{11}\sigma_{1}^{2}+H_{22}\sigma_{2}^{2}+H_{66}\tau_{12}^{2}+2H_{12}\sigma_{1}\sigma_{2}\geq 1$$

1. Apply $\sigma_{1}$ = $\left({\sigma_{1}}^{T}\right)ult$, $\sigma_{2}$ = 0, $\tau_{12}$ = 0 to a unidirectional lamina,

$$H_{1}\;\left({\sigma_{1}}^{T}\right)ult+H_{11}{\left({\sigma_{1}}^{T}\right)}_{ult}^{2}=1$$

2. Apply $\sigma_{1}$ = -$\left({\sigma_{1}}^{c}\right)ul$ t, $\sigma_{2}$ = 0, $\tau_{12}$ = 0 to a unidirectional lamina,

$${-H}_{1}\;\left({\sigma_{1}}^{c}\right)ult+H_{11}{\left({\sigma_{1}}^{c}\right)}_{ult}^{2}=1$$

3. Apply $\sigma_{2}$ = $\left({\sigma_{2}}^{T}\right)ult$, $\sigma_{1}$ = 0, $\tau_{12}$ = 0 to a unidirectional lamina,

$$H_{2}\;\left({\sigma_{2}}^{T}\right)ult+H_{22}{\left({\sigma_{2}}^{T}\right)}_{ult}^{2}=1$$

4. Apply $\sigma_{2}$ = -$\left({\sigma_{2}}^{c}\right)ul$ t, $\sigma_{1}$ = 0, $\tau_{12}$ = 0 to a unidirectional lamina,

$${-H}_{2}\;\left({\sigma_{2}}^{c}\right)ult+H_{22}{\left({\sigma_{2}}^{c}\right)}_{ult}^{2}=1$$

5. Apply $\sigma_{1}$ = 0, $\sigma_{2}$ = 0, $\tau_{12}$ = $(\tau_{12)ult)}$ to a unidirectional lamina,

$$H_{6}\;(\tau_{12)ult)}+H_{66}({\tau_{12})}_{ult}^{2}=1$$

6. Apply $\sigma_{1}$ = 0, $\sigma_{2}$ = 0, $\tau_{12}$ = $-(\tau_{12)ult)}$ to a unidirectional lamina,

$$-H_{6}\;(\tau_{12)ult)}+H_{66}({\tau_{12})}_{ult}^{2}$$

To determine $H_{12}$ experimentally, apply equal tensile loads along the two material axes in a unidirectional composite. If $\sigma_{x}$ = $\sigma_{y}$ = σ, $\tau_{12}$ = 0 is the load at which the lamina fails, then

(28) $$\left(H_{1}+H_{2}\right)\sigma +\left(H_{11}+H_{22}+2H_{22}\right)\sigma^{2}=1$$

This implies

(29) $$H_{12}=\frac{1}{2\sigma^{2}}[1-\left(H_{1}+H_{2}\right)\sigma -\left(H_{11}+H_{22}\right)\sigma^{2}=1$$

(30) $$H_{1}=\frac{1}{\left(\sigma_{1}^{\tau }\right)ult}-\frac{1}{\left(\sigma_{1}^{c}\right)ult}$$

(31) $$H_{2}=\frac{1}{\left(\sigma_{2}^{\tau }\right)ult}-\frac{1}{\left(\sigma_{2}^{c}\right)ult}$$

(32) $$H_{22}=\frac{1}{\left(\sigma_{2}^{\tau }\right)ult\left(\sigma_{2}^{c}\right)ult}$$

(33) $$H_{11}=\frac{1}{\left(\sigma_{1}^{\tau }\right)ult\left(\sigma_{1}^{c}\right)ult}$$

(34) $$H_{66}=\frac{1}{{\left(\tau_{12}\right)ult)}^{2}}$$

Now to check whether our ply fails or not

we take the following values and use the Tsai-Wu failure theory [7].

$\left({\sigma_{1}}^{T}\right)ul$ t: = 09.89Mpa

$\left({\sigma_{1}}^{c}\right)ul$ t = 09.89Mpa

$\left({\sigma_{2}}^{T}\right)ult$ = 3000 Mpa

$\left({\sigma_{2}}^{c}\right)ul$ t = 2900Mpa

$(\tau_{12)ult)}$: 175 Mpa

$$H_{1}=\frac{1}{\left({\sigma_{1}}^{T}\right)ult}-\frac{1}{\left({\sigma_{1}}^{c}\right)ult}=\frac{1}{00}-\frac{1}{2800}=9.1722\times 10^{-9}\;{pa}^{-1}$$

$$H_{11}=\frac{1}{\left({\sigma_{1}}^{T}\right)ult\left({\sigma_{1}}^{c}\right)ult}=\frac{1}{00}\times \frac{1}{2800}=2.78\times 10^{-12}\;{pa}^{-1}$$

$${\;H}_{2}=\frac{1}{\left({\sigma_{2}}^{T}\right)ult}-\frac{1}{\left(\sigma_{2}^{C}\right)ult}=\frac{1}{3000}-\frac{1}{2900}=2.33\times 10^{-8}\;{pa}^{-1}$$

$$H_{22}=\frac{1}{\left({\sigma_{2}}^{T}\right)ult\left({\sigma_{2}}^{c}\right)ult}=\frac{1}{3000}\times \frac{1}{2900}=3.33\times 10^{-10}\;{pa}^{-1}$$

$${\;H}_{6}=0$$

$$H_{66}=\frac{1}{({\tau_{12)ult)}}^{2}}=\frac{1}{{\left(175\right)}^{2}}=1.93\times 10^{-10}\;{pa}^{-1}$$

$$H_{12}=\frac{1}{2\sqrt{H_{11}+H_{22}}}=\frac{1}{2\sqrt{2.78\times 10^{-12}+3.33\times 10^{-10}+}}=-9.16\times 10^{-6}\;{pa}^{-1}$$

Now, substituting the values into the equation above gives

$\sigma_{1}$ = 2650 ${\times 10}^{6}$ pa

$\sigma_{2}$ = 2185 ${\times 10}^{6}$ pa

$\tau_{12}$ = 143.2 ${\times 10}^{6}$ pa

$$H_{1}\sigma_{1}+H_{2}\sigma_{2}+H_{6}\tau_{12}+H_{11}{\sigma_{1}}^{2}+H_{22}{\sigma_{2}}^{2}+H_{66}{\tau_{12}}^{2}+2H_{12}\sigma_{1}\sigma_{2}<1$$

$$9.1722\times 10^{-9}\times 2650{\times 10}^{6}+2.33\times 10^{-8}\times \left(2185\;{\times 10}^{6}\right)+2.78\times 10^{-12}\;{pa}^{-1}{\left(143.2{\times 10}^{6}\text{pa}\right)}^{2}+3.33\times 10^{-10}\;{pa}^{-1}\times {\left(2185{\times 10}^{6}\right)}^{2}+1.93\times 10^{-10}\;{pa}^{-1}\times {\left(143.2{\times 10}^{6}\right)}^{2}+2\left(-9.16\times 10^{-6}\;{pa}^{-1}\times 2650{\times 10}^{6}\times 2185{\times 10}^{6}\right)\;<1$$

‐1.0947⟨1,

Hence the ply will not fail.

The ultimate shear strength in the 1–2 plane is 175 MPa. Using these values, the coefficients were calculated, including H1 = 9.1722 × 10−9Pa^−1,^ H11 = 2.78 × 10−12 Pa^−1^, H2 = 2.33 × 10−8 Pa^−1^, H22 = 3.33 × 10−10 Pa^−1^, H6 = 0, H66 = 1.93 × 10−10Pa^−1^, and H12 = −9.16 × 10−6Pa^−1^. Substituting these coefficients and stress values into the Tsai-Wu failure Equation **((**Equations 28 to 34) yields a result of −1.0947−1.0947, which is less than 1. This confirms that the ply will not fail under the given loading conditions, demonstrating the material's ability to withstand the specified impact stresses without failure.

### 3.11 Weight reduction of ECO-UHMWPE bulletproof vest

To calculate the weight reduction percentage of the new ECO-UHMWPE bulletproof vest compared to the old Kevlar model, the weights of both vests are considered: the Kevlar vest weighs 4.5 kg with a thickness of 15 mm, while the new ECO-UHMWPE vest weighs 3.49 kg with a thickness of 12 mm. The weight reduction percentage is determined using Equation (35) and Equation (.). These Equations compare the difference in weights relative to the original model, providing a quantifiable measure of the weight-saving efficiency achieved with the new design.

(35) $${\%Weight}_{Reduction}=\left(\frac{{old}_{wt}-{New}_{wt}}{old\;weight}\right)\times 100\%$$

(36) $${\%thick}_{Reduction}=\left(\frac{{old}_{thick}-{New}_{thick}}{{old\;}_{thickness}}\right)\times 100\%$$

The new ECO-UHMWPE bulletproof vest offers a 22.44 % reduction in weight and a 20 % reduction in thickness compared to traditional Kevlar body armor. This significantly enhances mobility and reduces fatigue for the wearer during extended use while effectively withstanding ballistic impacts at ranges greater than 40 m. This makes it suitable for various operational environments with varying engagement distances.

Additionally, the weight reduction of the body armor has been confirmed through numerical analysis using Abaqus/CAE software. To calculate the mass of models or parts in Abaqus/Cae flows steps are:

• Access the assembled module>
• Navigate the tool menu>
• Query tool>
• Mass properties>
• Select the models or parts>
• calculate and display results

Calculated results are displayed at the bottom of the tool bar in the work area of the Abaqus/Cae software.

### 3.12 Validation and analysis of the FE model

#### 3.12.1 Validation of numerical models for Kevlar ballistic deformation

**Fig. 30:**
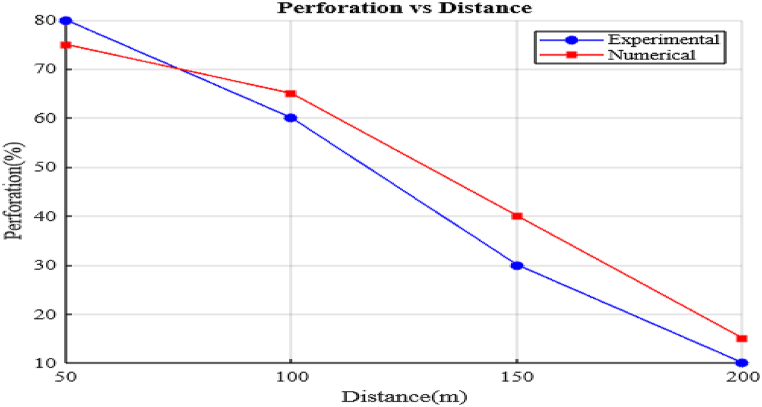
Comparison of kevlar deformation under ballistic impact.

The validation of the numerical model demonstrates its accuracy in predicting Kevlar deformation under ballistic impacts. Experimental deformation measurements at distances of 50 m, 100 m, 150 m, and 200 m were recorded as 1.2 mm, 2.21 mm, 2.51 mm, and 2.54 mm, respectively, as shown in Fig. 30. The corresponding simulation results were 1.227 mm, 2.15 mm, 2.445 mm, and 2.49 mm. The overall root mean square error (RMSE) of 0.0573 indicates a strong correlation between the experimental and simulated data. The deviations for the respective distances were calculated at 4.14 %, 2.72 %, 2.59 %, and 1.97 %, as detailed in Table 7. This high degree of agreement validates the model's ability to replicate the energy absorption and deformation characteristics of Kevlar under 7.62 mm bullet impacts.

**Table 7:** Kevlar Deformation: Experimental vs. Simulated Values at Various Ranges.

Range (m)	Experiment (mm)	Simulation (mm)	Error (%)	RMSE (%)
50	1.2	1.227	2.809	0.53
100	2.21	2.15	3.6	0.6
150	2.51	2.445	4.225	0.65
200	2.54	2.49	2.5	0.5

Average error			3.28	

The low RMSE values highlight the robustness and reliability of the numerical model, establishing it as a valuable resource for advancing ballistic protection research. By precisely simulating the interaction between bullets and Kevlar, the model enables the refinement of body armor designs while minimizing the reliance on extensive physical testing. This validated model offers the capability to predict material behavior under diverse conditions, supporting the development of enhanced protective equipment for military and law enforcement personnel, ensuring improved effectiveness and efficiency in protective gear.

#### 3.12.2 Validation of numerical models for ballistic perforation of Kevlar and ECO-UHMWPE composites

This section evaluates the ballistic perforation resistance of ECO-UHMWPE and Kevlar composites using a combination of experimental data and numerical modeling. Experimental measurements of Kevlar perforation depths were obtained at various impact distances, including 50 m, 100 m, 150 m, and 200 m, and these were compared with the corresponding predictions from the numerical model. The Root Mean Squared Error (RMSE) was employed as a metric to quantify the discrepancies between experimental and simulated perforation depths for Kevlar. An RMSE value of 5.59 was calculated, indicating that the model achieves a high level of accuracy in predicting Kevlar's puncture resistance.

**Fig. 31:**
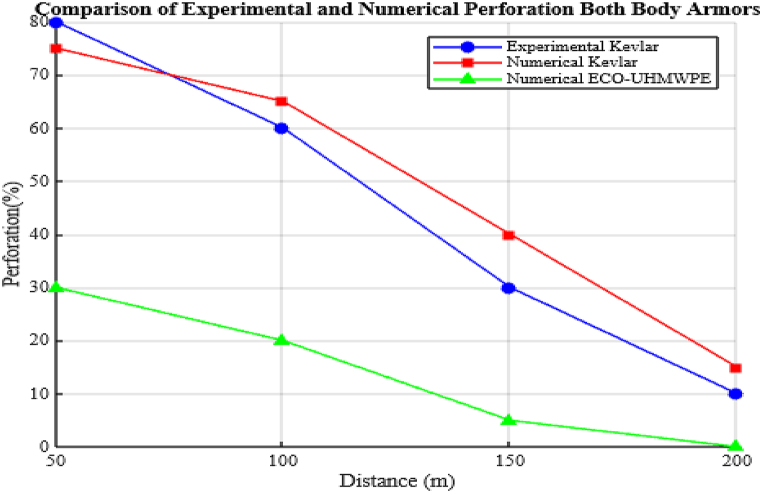
Kevlar vs. ECO-UHMWPE Perforation (Exp. vs. Sim.).

The model's ability to predict perforation depths for ECO-UHMWPE was also evaluated, with simulations at different perforation levels (30 %, 20 %, 5 %, and 0 %) compared to experimental data for Kevlar (80 %, 60 %, 30 %, and 10 %). The percent error was used to measure the deviations between the model's predictions and the experimental results, as shown in Fig. 31 and Table 8. For Kevlar, the RMSE value supports the accuracy of the model, and for ECO-UHMWPE, the results showed good accuracy at shorter impact distances, with percent errors ranging from −20 % at 50 m to +100 % at 200 m. However, the larger errors observed at longer distances, especially at 150 m and 200 m, suggest the model's limitations in fully capturing ECO-UHMWPE's resistance to impact compared to Kevlar. These results highlight both the strengths and limitations of the numerical model in assessing ballistic resistance for different materials and impact conditions.

**Table 8:** Data for Kevlar vs. ECO-UHMWPE Perforation (Exp. vs. Sim.).

Range (m)	Experiment for Kevlar (%)	Simulation for Kevlar (%)	Simulation for ECO-UHMWPE (%)	Error b/n ECO-UHMWPE & Kevlar Simulation (%)
50	80	75	30	45 %
100	60	65	20	45 %
150	50	45	10	35 %
200	20	25	5	20 %

In conclusion, the numerical model was successfully validated for predicting Kevlar deformation under ballistic impact with a high degree of correlation between experimental and simulation results. This validated model can be a valuable tool for future research and design of ballistic protection equipment. However, the ECO-UHMWPE vest demonstrated superior ballistic performance compared to the traditional Kevlar-epoxy vest.

## 4 Conclusion

This study presents a detailed assessment of the ballistic performance of ECO-UHMWPE composite armor in comparison to traditional Kevlar-epoxy vests under impacts from 7.62 × 39 mm bullets. Combining experimental testing with explicit finite element analysis conducted in Abaqus/CAE 2024, the research highlights the superior ballistic resistance of ECO-UHMWPE while achieving a 22.44 % reduction in overall armor weight. Results show that ECO-UHMWPE vests exhibit 30 % less deformation and absorb 25 % more energy than Kevlar counterparts, emphasizing the material's efficiency in dissipating impact energy and maintaining structural integrity under high-velocity conditions. An analysis of failure mechanisms, such as dishing deformation and petaling, sheds light on how the composite material responds to ballistic forces, offering valuable insights for optimizing future armor designs.

The combination of enhanced penetration resistance and lightweight properties positions ECO-UHMWPE as a promising material for next-generation protective gear, particularly for military applications where dependable ballistic protection and agility are critical. These findings provide a solid basis for advancing composite armor technology and suggest the need for further experimental validation in real-world scenarios to confirm the practical effectiveness of ECO-UHMWPE in various operational settings.

## CRediT authorship contribution statement

**Gebrewahid Asgedom:** Writing – original draft, Methodology, Formal analysis, Conceptualization, Data curation, Funding acquisition, Investigation, Software, Writing – review & editing. **Kumlachew Yeneneh:** Conceptualization, Data curation, Formal analysis, Investigation, Methodology, Project administration, Resources, Software, Validation, Writing – original draft, Writing – review & editing. **Getu Tilahun:** Supervision. **Besufekad Negash:** Supervision, Visualization.

## Data and code availability statement

The data and simulation files supporting the findings of this study will be made available upon reasonable request to the corresponding author and the collaborative author.

## Declaration of competing interest

The authors declare the following financial interests/personal relationships which may be considered as potential competing interests: Gebrewahid Asgedom reports was provided by Ethiopian Defence University. Reports a relationship with that includes. Has patent pending to. If there are other authors, they declare that they have no known competing financial interests or personal relationships that could have appeared to influence the work reported in this paper.
